# Identification of molecular subtypes and a prognostic signature based on machine learning and purine metabolism-related genes in breast cancer

**DOI:** 10.1097/MD.0000000000042288

**Published:** 2025-05-23

**Authors:** Wei Huang, Pengting Tang, Jingjie Mei, Zhan Zhang, Gang Lu

**Affiliations:** a Department of Surgery, Ninghai Maternal and Child Health Hospital, Ningbo, Zhejiang, China.

**Keywords:** breast cancer, immune landscape, immunotherapy, nomogram, purine metabolism

## Abstract

Breast cancer (BC), one of the most prevalent malignant tumors worldwide, lacks efficacious diagnostic biomarkers and therapeutic targets. This study harnesses multi-omics data to identify novel purine metabolism-related genes (PMRG) as potential biomarkers and risk signatures. Univariate Cox regression was employed to assess the correlation between PMRGs and BC patient prognosis, while a Lasso Cox model was constructed to derive a prognostic signature. Gene set enrichment analysis was utilized to investigate functional differences. Kaplan–Meier survival curves were plotted to examine overall survival disparities between these 2 risk groups, with further exploration into the relationship between the prognostic signature, immune landscape, and drug sensitivity. Ultimately, a predictive nomogram was developed based on these findings. BC patients were stratified into 2 distinct molecular subtypes with significantly different prognoses using the identified PMRG-based signature, which comprised 17 PMRGs. This signature emerged as an independent prognosticator for BC and was integrated into a nomogram along with age, chemotherapy/radiotherapy treatment history, and clinical staging to accurately predict patient outcomes. Moreover, the signature showed associations with the tumor immune microenvironment and drug responsiveness, where lower-risk patients exhibited increased chemotherapeutic sensitivity, immune scores, and decreased tumor purity. Gene set enrichment analysis highlighted significant activation in pathways such as the complement and coagulation cascades, ribosome biogenesis, MAPK signaling, cAMP signaling, and drug metabolism pathways in the low-risk group. The derived PMRG-based signature holds promise for predicting the prognosis of BC patients and guiding their clinical management, including immunotherapy interventions.

## 1. Introduction

Breast cancer (BC) remains a significant global health challenge, representing one of the most prevalent cancers affecting women worldwide.^[1]^ Despite advancements in diagnosis and treatment strategies, the heterogeneity of BC tumors and their varied responses to therapy underscore the necessity for personalized approaches to prognosis and treatment selection.^[2,3]^ One promising avenue for enhancing our understanding of BC prognosis lies in exploring the intricate interplay between genetic factors and metabolic pathways.^[4]^

BC is a complex disease characterized by aberrant cellular growth and proliferation. Its etiology involves a multitude of genetic and environmental factors, leading to diverse molecular subtypes with distinct clinical behaviors.^[5]^ Among the myriad molecular pathways implicated in BC pathogenesis, alterations in purine metabolism have emerged as a focal point of interest.^[6]^ Purines, comprising adenine and guanine, serve as essential components of DNA, RNA, and various signaling molecules. Dysregulation of purine metabolism can perturb cellular homeostasis, contributing to tumor initiation, progression, and therapeutic resistance.^[7,8]^

The exploration of purine metabolism in the context of BC holds promise for elucidating novel prognostic biomarkers and therapeutic targets.^[9]^ However, the intricate network of genes involved in purine metabolism and their specific roles in BC progression remain incompletely understood. Therefore, this study aims to construct a comprehensive prognostic model based on purine metabolism-related genes (PMRG) to improve risk stratification and clinical management of BC patients. By integrating genomic data with clinical outcomes, we seek to identify key genetic signatures associated with disease progression and patient survival, ultimately paving the way for personalized therapeutic interventions and improved prognostic accuracy in BC care, Data S1, Supplemental Digital Content, https://links.lww.com/MD/O990.

## 2. Materials and method

### 2.1. PMRGs and datasets

From the Molecular Signatures Database (MSigDB, accessible at https://www.gsea-msigdb.org/gsea/msigdb/index.jsp), a collection of 166 PMRGs was retrieved (Table S1, Supplemental Digital Content, https://links.lww.com/MD/O981). This study incorporated a total of 1113 breast invasive carcinoma (BRCA) samples and 113 normal tissue specimens sourced from the Cancer Genome Atlas (TCGA). Of these, 869 BRCA cases with complete clinical-pathological characteristics and follow-up data were utilized for prognosis assessment and model construction. Additionally, transcriptomic profiles along with corresponding clinicopathological information from 3406 BC patients were obtained from the GSE96058 dataset within the Gene Expression Omnibus database (https://www.ncbi.nlm.nih.gov/geo/).

### 2.2. Tumor categorization

PMRGs associated with BC prognosis were assessed via univariate Cox regression analysis, and those with *P*-values <.05 underwent consensus clustering analysis. This process was executed using the ConsensusClusterPlus package,^[10]^ applying the Partitioning Around Medoids algorithm and the “pearson” distance metric. Kaplan–Meier survival curves were utilized to evaluate the differences in prognosis among the resulting clusters.

### 2.3. Development of a PMRG-based risk signature

To establish a risk signature for PMRGs, Lasso Cox regression analysis was performed using the glmnet package, aimed at dimensionality reduction of prognostically relevant PMRGs. A risk score was computed according to the following formula: RiskScore = ∑(Coefficient_i × Exp_i), where Coefficient_i denotes the coefficient assigned to gene i and Exp_i represents its corresponding expression level. Based on this risk score, patients were stratified into 2 subgroups: low-risk (≤median risk score) and high-risk (≥median risk score). Kaplan–Meier curves were applied to compare the prognosis difference between the high- and low-risk groups, and the predictive performance of the risk score was evaluated through the receiver operating characteristic (ROC) curve analysis.

### 2.4. Gene set enrichment analysis (GSEA)

To elucidate the biological processes and pathways linked to the established risk signature, we conducted GSEA to assess dynamic activities and pathways enriched across different risk subclasses. Initially, differential expression analysis was carried out between the groups using the edgR package,^[11]^ generating a list of genes with their respective log-fold changes. Subsequently, gene ontology (GO) annotations and Kyoto Encyclopedia of Genes and Genomes pathways were subjected to GSEA utilizing the clusterProfiler package.^[12]^

### 2.5. Assessment of tumor immune microenvironment

We leveraged the IOBR package^[13]^ to evaluate the tumor immune microenvironment in BC, contrasting differences in the infiltration levels of 22 immune cell types between the high- and low-risk groups. Correlations between the risk score and its constituent genes with immune cell infiltration patterns were also analyzed. Furthermore, the ESTIMATE algorithm was employed to quantify stromal and immune scores, as well as tumor purity. Additionally, the immunophenoscore was calculated to characterize BC immunophenotypes, indicative of potential responses to anti-cytotoxic T-lymphocyte-associated protein 4 (anti-CTLA-4) and anti-programmed cell death protein 1 (anti-PD-1) antibody therapies.

### 2.6. Drug sensitivity assessment

Utilizing the pRRophetic package,^[14]^ we assessed drug sensitivity in BC patients and investigated correlations between the risk score and its component gene expressions with drug sensitivity, comparing differences in drug response between the high- and low-risk categories.

### 2.7. Construction and evaluation of nomogram

Through univariate and multivariate Cox regression analyses, we identified prognostic factors for BC and selected independent predictors (*P* < .05) to construct a nomogram. Model performance was appraised using calibration curves, ROC curves, and decision curves to validate the predictive accuracy and clinical utility of the nomogram.

## 3. Results

### 3.1. Mutation, expression, and prognostic characteristics of PMRGs

**Figure 1.**
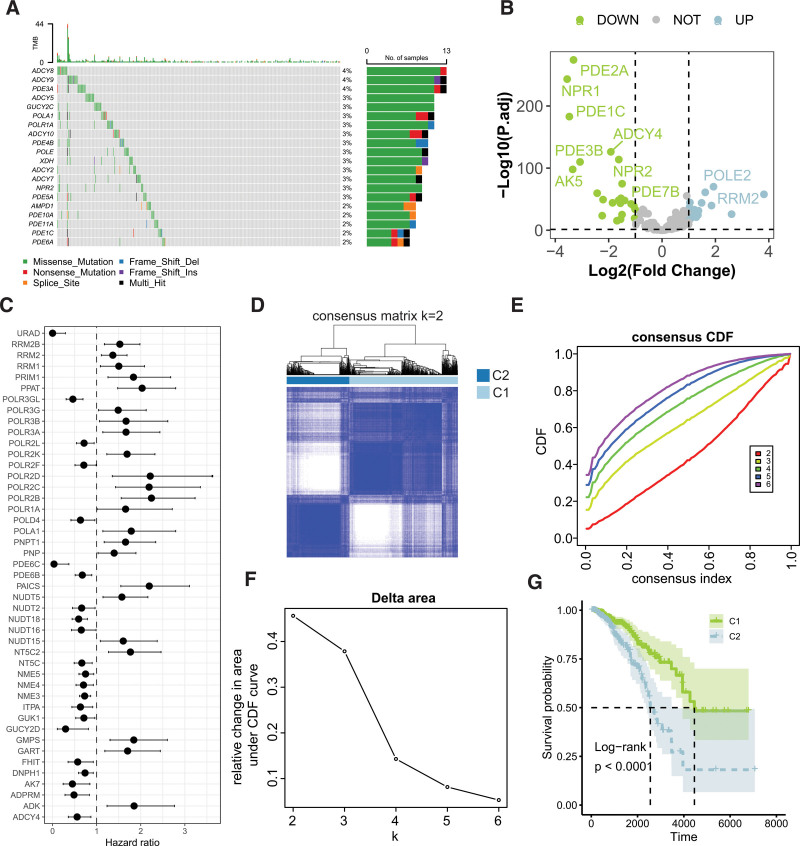
Molecular subtyping of breast cancer based on PMRGs associated with prognosis. (A) Somatic mutation landscape of PMRGs. (B) Volcano plot illustrating differential expression of PMRGs in breast cancer. (C) Hazard ratios of PMRGs relevant to prognosis. (D–F) Consensus clustering of breast cancer based on the selected PMRGs with prognostic significance. (G) Kaplan–Meier survival analysis comparing the prognostic differences among the PMRG-derived molecular subtypes. PMRG = purine metabolism-related genes.

Figure 1A illustrates somatic mutations in PMRGs, wherein the top 3 genes most frequently mutated are ADY8, ADCY9, and PDE3A. Differential expression analysis identified 42 PMRGs that were aberrantly expressed in BC, with 18 significantly overexpressed and 24 underexpressed (Fig. 1B). Univariate Cox regression analysis revealed that 46 PMRGs were associated with BC prognosis, among which 22 were correlated with favorable outcomes, while the remaining 24 were associated with poorer prognosis (Fig. 1C; Table S2, Supplemental Digital Content, https://links.lww.com/MD/O981). Based on these prognostically relevant PMRGs, consensus clustering analysis discerned 2 molecular subtypes of BC (Fig. 1D–F), with a statistically significant difference in survival observed between these subtypes (Fig. 1G).

### 3.2. Derivation of a PMRG-informed risk signature

**Figure 2.**
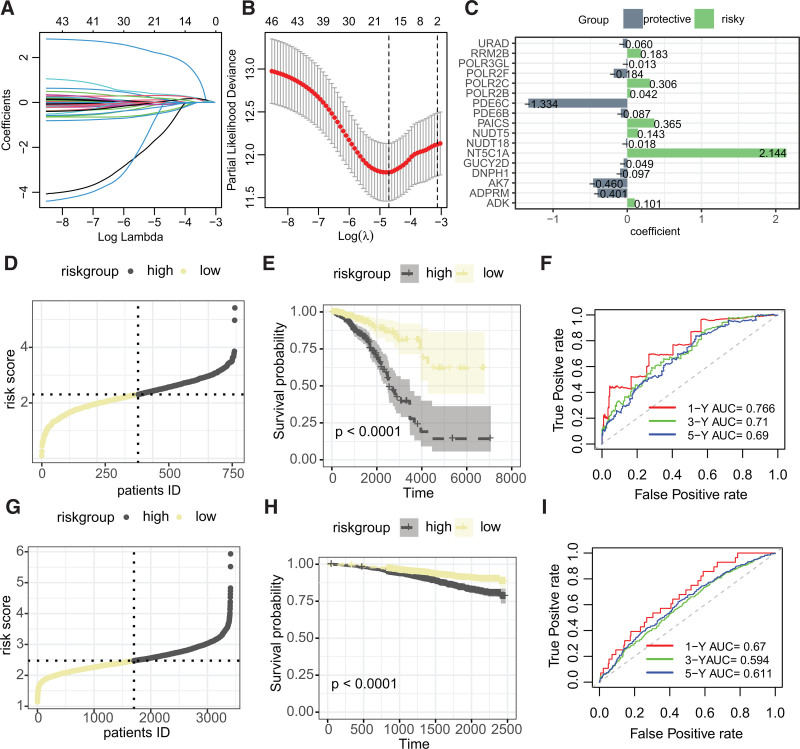
Construction and evaluation of the PMRG-derived breast cancer risk signature. (A and B) Lasso Cox regression analysis for screening and dimensionality reduction of PMRGs associated with prognosis. (C) Coefficients of the 17 PMRGs in the riskscore. (D–F) Stratification of the TCGA–BRCA cohort into high- and low-risk groups based on the riskscore followed by Kaplan–Meier survival analysis and receiver operating characteristic (ROC) curve analysis. (G–I) Similar stratification, survival analysis, and ROC curve analysis for the GSE96058 cohort using the riskscore. BRCA = breast invasive carcinoma, PMRG = purine metabolism-related genes, TCGA = The Cancer Genome Atlas.

Further, lasso Cox regression analysis was employed for dimensionality reduction, leading to the selection of 17 PMRGs for constructing a risk signature (Fig. 2A and B), with Fig. 2C presenting the coefficients of each gene in the model. Riskscores were calculated for both the TCGA–BRCA and GSE96058 cohorts using the derived formula and dichotomized into high- and low-risk groups by the median value (Fig. 2D and G). Kaplan–Meier survival analysis demonstrated that the high-risk group had significantly worse prognosis compared to the low-risk group (Fig. 2E and H). ROC curves showed that the areas under the curve (AUC) for riskscore predicting 1-, 3-, and 5-year overall survival were 0.766, 0.71, and 0.69 in the TCGA–BRCA cohort (Fig. 2F), respectively, and in the GSE96058 cohort, the AUCs were 0.67, 0.594, and 0.611 for the same time points (Fig. 2I).

### 3.3. Clinical pathological and somatic mutation features between high and low-risk groups

**Figure 3.**
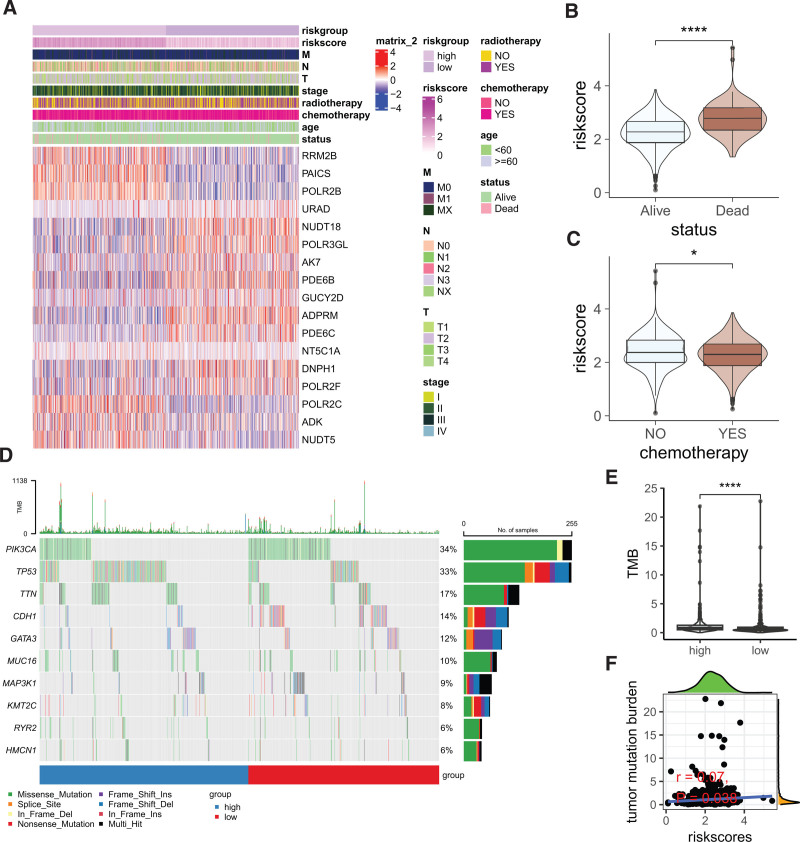
Association of the PMRG-derived risk signature with clinical, mutational, and treatment features. (A) Heatmap displaying the expression of PMRGs composing the risk signature alongside annotated clinical-pathological information. (B) Comparison of the riskscore between dead and alive patient groups. ****P < .0001. (C) Comparison of the riskscore between patients receiving chemotherapy and those who did not. *P < .05. (D) Oncoplot showing somatic mutations across high and low-risk groups. ****P < .0001. (E) Comparison of tumor mutation burden (TMB) between high and low-risk groups. (F) Scatterplot illustrating the correlation between the riskscore and TMB. PMRG = purine metabolism-related genes.

Figure 3A presents the expression pattern of risk signature-related genes annotated with their corresponding clinical pathological characteristics. Notably, patients who succumbed to the disease had significantly higher riskscores compared to survivors (Fig. 3B). Additionally, patients receiving chemotherapy exhibited lower riskscores compared to those not treated with chemotherapy (*P* < .01, Fig. 3C); however, no other significant differences in riskscores were observed among various clinical pathological subgroups. Figure 3D depicts the differences in somatic mutations between the high- and low-risk groups. The high-risk group had a significantly higher tumor mutation burden compared to the low-risk group (Fig. 3E). Moreover, a positive correlation was found between the riskscore and tumor mutation burden (*R* = 0.07, *P* = .038, Fig. 3F), indicating that the riskscore is indeed related to the mutational load of the tumor.

### 3.4. Association of PMRGs with chemotherapy sensitivity

**Figure 4.**
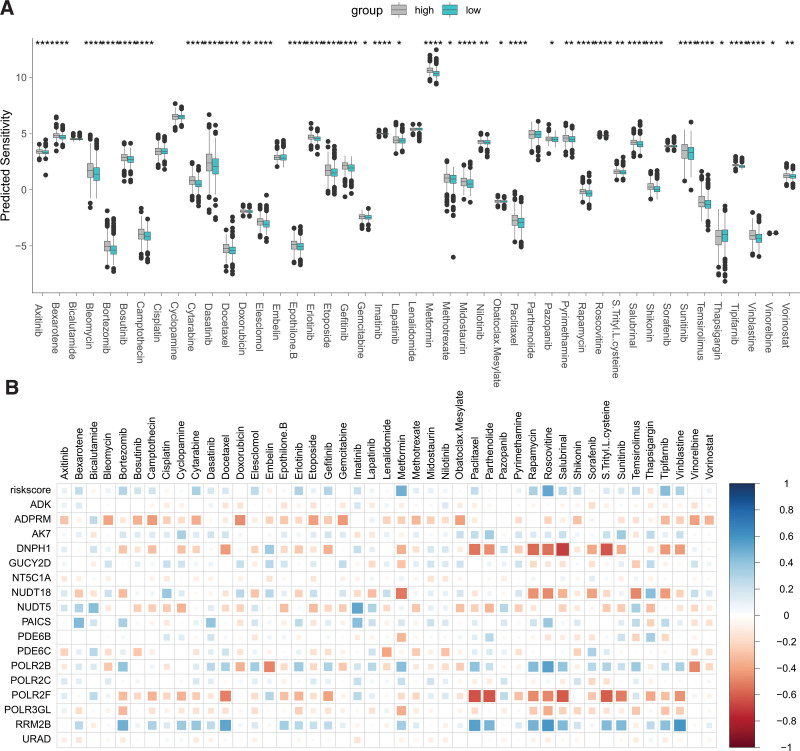
Relationship between the PMRG-derived risk signature and drug sensitivity. (A) Comparison of drug sensitivity between high and low-risk groups. *P < .05, **P < .01, ***P < .001, ****P < .0001. (B) Heatmap representing correlations between the riskscore and PMRG gene expression with drug sensitivity. PMRG = purine metabolism-related genes.

We investigated the relationship between PMRGs and drug sensitivity, revealing that the low-risk group displayed enhanced sensitivity to 37 drugs, including axitinib, bexarotene, and bleomycin, compared to the high-risk group (Fig. 4A). Correlation analysis indicated that the riskscore positively correlated with predicted sensitivity to the majority of drugs (Fig. 4B). Notably, ADPRM, POLR2F, and POLR2GL negatively correlated with the predicted sensitivity of most drugs, whereas RRM2B, PAICS, and AK7 showed a negative association (Fig. 4B). These findings suggest a significant connection between PMRGs and chemotherapy sensitivity in BC, potentially implicating them as targets for overcoming drug resistance.

### 3.5. PMRGs and tumor immune microenvironment

**Figure 5.**
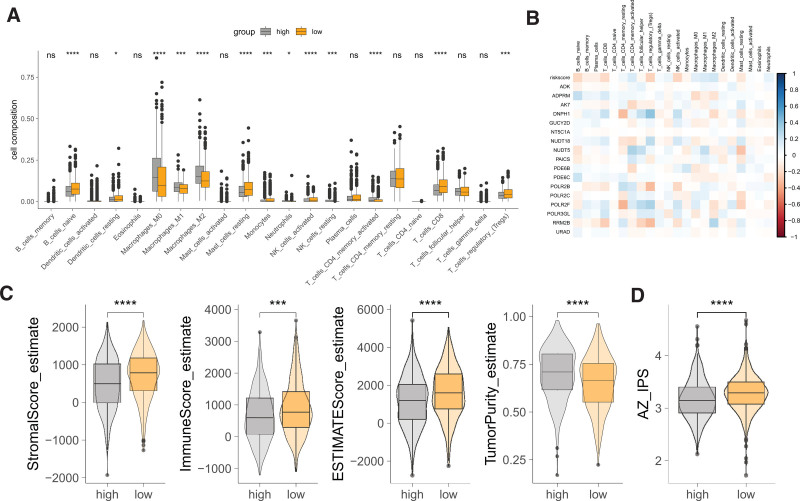
Analysis of the relationship between PMRGs and the tumor immune microenvironment. (A) Comparison of infiltration levels of 22 immune cell types between high and low-risk groups. ns: not significant, *P < .05, **P < .01, ***P < .001, ****P < .0001. (B) Heatmap depicting correlations between the riskscore and its constituent genes with immune cell infiltration. (C) Comparison of stromalscore, immunescore, estimatescore, and tumor purity between high and low-risk groups. ****P < .0001. (D) Comparison of infiltrating progenitor score (IPS) between high and low-risk groups. ****P < .0001. IPS = immunophenoscore, PMRG = purine metabolism-related genes.

Compared to the high-risk group, the low-risk group exhibited elevated infiltration levels of B cells, dendritic cells, mast cells, monocytes, NK cells, CD8+ T cells, and tregs, but reduced infiltration of macrophages and CD4+ T cells (Fig. 5A). The riskscore inversely correlated with B cells, CD8+ cells, tregs, and positively correlated with macrophages and CD4+ cells (Fig. 5B). Moreover, individual genes comprising the riskscore showed distinct significant correlations with immune cell infiltrates. The low-risk group had higher stromalscore, immunescore, estimatescore, and lower tumor purity compared to the high-risk group (Fig. 5C). Additionally, the low-risk group presented with a higher immunophenoscore compared to the high-risk group (Fig. 5D), collectively suggesting a substantial link between the riskscore and its component genes with the tumor immune microenvironment.

### 3.6. Gene expression patterns between high and low-risk groups

**Figure 6.**
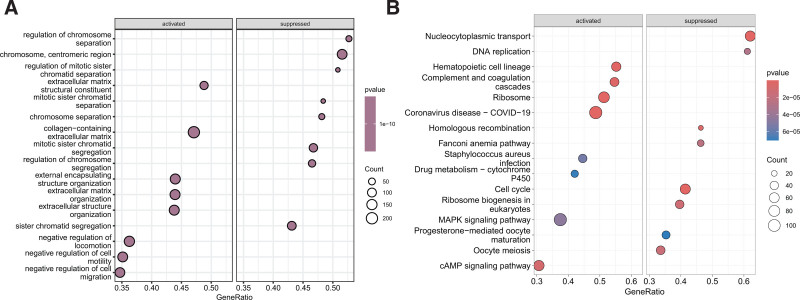
Gene expression profiling distinguishing high and low-risk groups. (A) Gene ontology enrichment analysis of genes differentially expressed between high and low-risk groups. (B) Kyoto Encyclopedia of Genes and Genomes (KEGG) pathway enrichment analysis of genes differentially expressed between high and low-risk groups.

GSEA uncovered that in the low-risk group, extracellular matrix organization, cell motility, and cell migration-related GO terms were significantly upregulated, while chromosome and chromatid segregation-related GO terms were downregulated (Fig. 6A). Compared to the high-risk group, several pathways, including the complement and coagulation cascades, ribosome, MAPK signaling, cAMP signaling pathway, and cytochrome P450-mediated drug metabolism, were significantly activated, whereas nucleocytoplasmic transport, DNA replication, Fanconi anemia pathway, and cell cycle-related pathways were suppressed (Fig. 6B).

### 3.7. Nomogram-based prediction of BC prognosis

**Figure 7.**
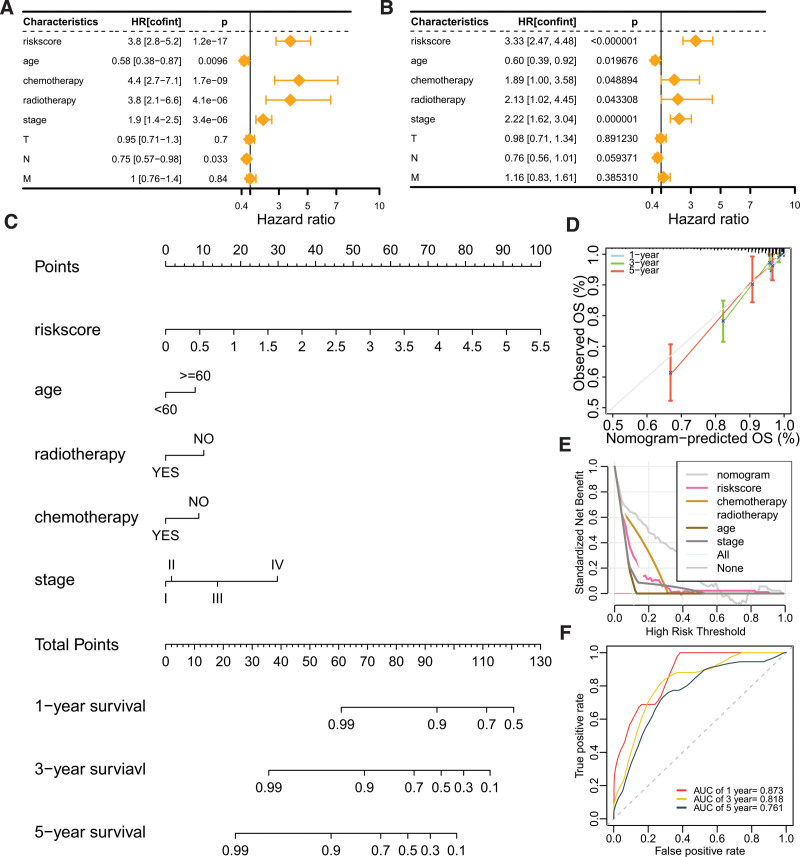
Development and evaluation of a breast cancer nomogram. (A) Univariate Cox regression analysis identifying prognostic factors for breast cancer. (B) Multivariate Cox regression analysis identifying independent prognostic factors for breast cancer. (C) Nomogram constructed for predicting 1-, 3-, and 5-year overall survival in breast cancer patients. (D) Calibration curve analysis of the nomogram. (E) Decision curve analysis demonstrating superior prognostic performance of the nomogram compared to other prognostic factors. (F) Time-dependent ROC analysis for the nomogram’s predictive accuracy of 1-, 3-, and 5-year overall survival rates. ROC = receiver operating characteristic.

Univariate Cox regression analysis identified riskscore, age, chemotherapy, radiotherapy, clinical stage, and N stage as prognostic factors for BC (Fig. 7A). Multivariate Cox regression analysis further confirmed that all these factors except N stage served as independent prognostic factors (Fig. 7B). Consequently, these independent prognostic factors were incorporated into a nomogram to estimate the 1-, 3-, and 5-year overall survival probabilities for BC patients (Fig. 7C). The nomogram demonstrated good calibration as the predicted overall survival closely followed the 45-degree line (Fig. 7D). Moreover, the standardized net benefit analysis showed that the nomogram provided superior prognostic performance over other features when predicting 1-year overall survival (Fig. 7E). ROC curve analysis revealed that the AUC values for the nomogram in predicting 1-, 3-, and 5-year overall survival were 0.873, 0.818, and 0.761, respectively (Fig. 7F), indicating its strong discriminative power.

## 4. Discussion

Risk signature based on PMRGs have been successfully constructed and demonstrated outstanding prognostic performance in colorectal cancer^[9]^; however, such models have not yet been developed for BC. In the present study, we have, for the first time, devised a BC risk signature comprising 17 PMRGs that effectively predicts prognosis in BC patients. This novel signature, when integrated into a nomogram along with age, radiotherapy, chemotherapy, and clinical staging data, can be clinically applied for accurate prognosis assessment in BC patients.

The correlation between PMRGs and BC prognosis offers additional targets for the development of targeted therapies in BC. ADK, a principal metabolic enzyme predominantly expressed in astrocytes that regulates adenosine homeostasis in the brain, has been shown to be aberrantly upregulated in gliomas.^[15]^ AK7, a member of the adenosine monophosphate (AMP) kinases, involved in adenosine monophosphate regeneration, is associated with poor prognosis in ovarian cancer upon low expression.^[16]^ PAICS, which exhibits abnormal overexpression in multiple tumors and correlates with unfavorable prognosis, modulates several signaling pathways to suppress tumor cell apoptosis, promote tumor cell proliferation, epithelial-mesenchymal transition, invasion, and metastasis.^[17]^ RRM2B plays a critical role in DNA replication, repair, and oxidative stress response; cancers with RRM2B amplification are characterized by DNA repair defects and oxidative stress-associated mutational profiles, and at least in BC, RRM2B amplification is linked to poorer clinical outcomes.^[18]^ POLR2F is upregulated in colorectal cancer and its expression is related to prognosis.^[19]^ A detailed summary of expression and functional studies^[15,16,19–58]^ of PMRGs biomarker genes was presented in Table 1.

**Table 1:** Summary of expression and functional studies of PMRGs biomarker genes in breast cancer.

Gene symbol	Gene name	Function	Expression
ADK	Adenosine kinase	miR-182-5p/ADK/SEMA5a axis involved in drug resistance^[20]^	Up-regulated^[21]^;
ADPRM	ADP-ribose/CDP-alcohol diphosphatase, manganese dependent	N/A	N/A
AK7	Adenylate kinase 7	Involved in the regulation of proliferation, invasion, and migration and associated with overall survival^[22,23]^;	Down-regulated^[22,23]^
DNPH1	2′-Deoxynucleoside 5′-phosphate N-hydrolase 1	DNPH1 deficiency enhance sensitivity to PARP inhibitors^[24]^; involved in cellular proliferation^[25,26]^;	Up-regulated^[27–29]^;
GUCY2D	Guanylate cyclase 2D, Retinal	N/A	N/A
NT5C1A	5′-Nucleotidase, cytosolic IA	Mediates gemcitabine resistance^[30]^;	Up-regulated^[30]^;
NUDT18	Nudix hydrolase 18	N/A	N/A
NUDT5	Nudix hydrolase 5	REGULATES proliferation^[31,32]^; involved in the regulation of profileration, invasion and chemosensitivity through the AKT/GSK-3β/β-catenin pathway^[33]^; activates the AKT/Cyclin D pathway^[34]^; promotes tumor metastasis^[35]^; predicts a poor survival^[36]^;	Up-regulated^[31–33]^
PAICS	Phosphoribosylaminoimidazole carboxylase and phosphoribosylaminoimidazolesuccinocarboxamide synthase	Predicts a poor survival^[37]^; involved in the regulation of profileration, invasion^[38–40]^;	Up-regulated^[37,40–42]^
PDE6B	Phosphodiesterase 6B	N/A	Up-regulated^[43]^
PDE6C	Phosphodiesterase 6C	N/A	Up-regulated^[43]^
POLR2B	RNA polymerase II Subunit B	Knockdown of POLR2B reduced xenograft tumor volume and prolonged the survival of nude mice^[44]^	Up-regulated^[44,45]^
POLR2C	RNA polymerase II Subunit C	Involved in the regulation of cell cycle and malignant proliferation^[46]^;	Up-regulated^[46,47]^
POLR2F	RNA polymerase II, I and III subunit F	Involved in drug resistance and differentiation^[48]^	Up-regulated^[47,49,50]^
POLR3GL	RNA polymerase III subunit GL	Involved in the regulation of cell cycle, differentiation and proliferation^[51,52]^; predicts unfavorable survival outcomes^[53]^;	Up-regulated^[52,53]^
RRM2B	Ribonucleotide reductase regulatory TP53 inducible subunit M2B	Associated with prognosis^[54–58]^; FOXO3/RRM2B and miR-942/RRM2B axis involved in the regulation of cancer cell proliferation^[59,60]^	Up-regulated^[55–58]^
URAD	Ureidoimidazoline (2-oxo-4-hydroxy-4-carboxy-5-) decarboxylase	Gene variants associated with increased cancer risk^[61]^	N/A

Metabolic reprogramming is implicated in drug resistance,^[59–61]^ and studies have revealed that IMPDH2-mediated purine metabolism promotes oxaliplatin resistance in colorectal cancer by suppressing caspase-dependent apoptosis.^[7]^ In diffuse midline gliomas, inhibition of purine salvage may contribute to overcoming treatment resistance.^[62]^ Notably, our findings demonstrate significant associations between PMRGs and drug sensitivity, thereby providing further potential targets for investigating cancer drug resistance. DNPH1, which participates in detoxification reactions, particularly against toxic substances like dinitrophenol, has been shown to significantly sensitize BRCA-deficient cancer cells to PARPi and hmdU treatments.^[22]^ Notably, metabolic alterations in breast cancer extend beyond purine metabolism. Previous studies by Jelski et al revealed paradoxical elevation of class I alcohol dehydrogenase (ADH) activity in serum of stage IV breast cancer patients, despite overall reduction of ADH/aldehyde dehydrogenase activities in tumor tissue.^[63,64]^ This suggests distinct regulatory mechanisms between localized and systemic metabolic adaptation. While our study focuses on purine metabolic genes in tumor microenvironment, the observed association between PMRGs and chemotherapy sensitivity may partially overlap with ethanol metabolism pathways. Particularly, ADH/aldehyde dehydrogenase-mediated acetaldehyde metabolism could influence drug detoxification processes.^[65,66]^ The stage-specific elevation of serum ADH I observed by Jelski et al warrants further investigation into potential crosstalk between purine and ethanol metabolic pathways in advanced cancers, which may synergistically contribute to therapeutic resistance.

The tumor immune microenvironment plays a pivotal role in the initiation, progression, and responsiveness to therapy in cancer.^[67–69]^ Our research suggests involvement of PMRGs in shaping the tumor microenvironment. Within this milieu, CD73 and CD39 are expressed by T lymphocytes, and their regulation is crucial for effective antitumor immune responses. Studies have indicated an association between ADK expression and the CD39/CD73/A2AR axis in colorectal cancer patients.^[70]^ Activation of A2AR contributes to immune suppression,^[71]^ while CD73 is overexpressed in tumor tissues and has been linked to metastasis, progression of colorectal cancer, and patient survival.^[72]^ DNA damage response is intimately connected with immune surveillance, particularly in preventing the accumulation of mutations that lead to tumorigenesis,^[73,74]^ and genes such as URAD and RRM2B, which participate in DNA synthesis and repair processes, may exert potential immunomodulatory functions.

The GSEA results provide critical insights into the biological mechanisms underpinning the prognostic value of the PMRG signature. The activation of complement and coagulation cascades in the low-risk group aligns with emerging evidence linking purine metabolism to immune regulation and thrombotic signaling in breast cancer. Adenosine, a key purine metabolite, modulates complement activation via A2A receptor signaling, which may suppress pro-tumorigenic inflammation and enhance immune surveillance.^[75]^ Similarly, the enrichment of MAPK and cAMP signaling pathways in low-risk patients reflects their roles in purine-mediated cellular homeostasis. For instance, cAMP signaling regulates purine biosynthesis through phosphorylation of rate-limiting enzymes like PRPS1/2,^[76]^ while MAPK activation downstream of purinergic receptors (e.g., P2Y/P2X) can inhibit proliferation and promote apoptosis in BC cells.^[77]^ The suppression of cell cycle pathways (e.g., DNA replication) in the low-risk group further supports the antiproliferative effects of balanced purine metabolism, as nucleotide availability directly governs S-phase progression.^[78]^ Conversely, the upregulation of drug metabolism pathways (e.g., cytochrome P450) in low-risk patients may explain their enhanced chemosensitivity, as purine analogs like 6-mercaptopurine rely on metabolic enzymes for activation.^[79]^ These findings collectively position the PMRG signature as a regulator of both tumor-intrinsic metabolic reprogramming and therapy-responsive pathways.

However, our study also has certain limitations. Firstly, the construction and evaluation of the risk signature based on a retrospective cohort design limit the generalizability of the model, hence, large-scale randomized clinical trials are imperative to substantiate the superiority of our model. Secondly, we have not conducted in vitro and in vivo experiments to thoroughly investigate the underlying mechanisms of our proposed signature.

## 5. Conclusion

In summary, our study has successfully constructed and evaluated a novel risk profile derived from PMRGs in BC, which is pertinent to BC prognosis, the tumor immune microenvironment, and drug sensitivity. This unique feature, when combined with clinical pathological characteristics, can be utilized for prognostic assessment in BC patients. These findings contribute to the advancement of personalized treatment strategies for BC patients, underscoring the importance of integrating molecular signatures with conventional clinicopathological parameters to enhance therapeutic precision and improve patient outcomes.

## Author contributions

**Conceptualization:** Wei Huang, Pengting Tang, Jingjie Mei, Zhan Zhang, Gang Lu.

**Data curation:** Wei Huang, Pengting Tang, Jingjie Mei, Zhan Zhang, Gang Lu.

**Formal analysis:** Wei Huang, Pengting Tang, Jingjie Mei, Zhan Zhang, Gang Lu.

**Funding acquisition:** Wei Huang, Pengting Tang, Jingjie Mei, Zhan Zhang, Gang Lu.

**Investigation:** Wei Huang, Pengting Tang, Jingjie Mei, Zhan Zhang, Gang Lu.

**Methodology:** Wei Huang, Pengting Tang, Jingjie Mei, Zhan Zhang, Gang Lu.

**Project administration:** Wei Huang, Pengting Tang, Jingjie Mei, Zhan Zhang, Gang Lu.

**Resources:** Wei Huang, Pengting Tang, Jingjie Mei, Zhan Zhang, Gang Lu.

**Software:** Wei Huang, Pengting Tang, Jingjie Mei, Zhan Zhang, Gang Lu.

**Supervision:** Wei Huang, Pengting Tang, Jingjie Mei, Zhan Zhang, Gang Lu.

**Validation:** Wei Huang, Pengting Tang, Jingjie Mei, Zhan Zhang, Gang Lu.

**Visualization:** Wei Huang, Pengting Tang, Jingjie Mei, Zhan Zhang, Gang Lu.

**Writing – original draft:** Wei Huang, Gang Lu.

**Writing – review & editing:** Wei Huang, Gang Lu.

## Supplementary Material


