# Supplementary material for: Identification of molecular subtypes and a prognostic signature based on machine learning and purine metabolism-related genes in breast cancer
Source: Medicine (Baltimore). 2025 May 23;104(21):e42288. doi: 10.1097/MD.0000000000042288 (PMC12114004; doi:10.1097/MD.0000000000042288)
Supplement: Supplementary file 1 [file medi-104-e42288-s001.docx]

#######################################################

### Breast Cancer Purine Metabolism Analysis Script ###

### Code for Peer Review ###

#######################################################

#------------------ Environment Setup -----------------

# Install required packages (if not already installed)

if (!require("BiocManager")) install.packages("BiocManager")

BiocManager::install(c("limma", "edgeR", "maftools", "ConsensusClusterPlus"))

# Load required libraries

library(limma)

library(edgeR)

library(maftools)

library(ConsensusClusterPlus)

library(survival)

library(survminer)

#----------------------Differential Expression Analysis------------------------------------

rm(list = ls())

library(limma)

library(tidyverse)

library(stringr)

library(edgeR)

library(dplyr)

library(gridExtra)

library(survival)

library(survminer)

library(forestplot)

library(ezcox)

library(forestmodel)

library(ggrepel)

library(xlsx)

#----Load Data---

load("exp_count.rda") # Load count data

load("exp_fpkm.rda") # Load FPKM data

load("geo_exp.rda") # Load validation dataset

tcga_clinical <- read.csv("tcga_clinical.csv", header = TRUE)

geo_clinical <- read.csv("geo_clinical.csv", header = TRUE)

geneset <- read.xlsx("purine metabolism-related genes.xlsx", sheetIndex = 2, header = TRUE) # Target gene set

#----Extract Target Gene Set Expression Matrix---

geneset <- geneset$Symbol

#----Get Target Gene Set Expression Matrix---

exp <- exp_count_gene_name[rownames(exp_count_gene_name) %in% geneset, ]

dim(exp)

#----Prepare Group Information---

group <- factor(rep(c("Normal", "Tumor"), times = c(113, 1113)),

levels = c("Normal", "Tumor"))

table(group)

design <- model.matrix(~0 + group)

colnames(design) <- levels(group)

row.names(design) <- colnames(exp_count_gene_name)

#----Differential Expression Analysis---

DGElist <- DGEList(counts = exp, group = group)

DGElist <- calcNormFactors(DGElist) # Normalization

#----Convert Data to logCPM and Fit Linear Model---

v <- voom(DGElist, design, plot = TRUE, normalize = "quantile") # logCPM conversion

fit <- lmFit(v, design) # Fit linear model

constrasts <- paste(rev(levels(group)), collapse = "-")

cont.matrix <- makeContrasts(contrasts = constrasts, levels = design)

fit2 <- contrasts.fit(fit, cont.matrix)

fit2 <- eBayes(fit2)

DEG <- topTable(fit2, coef = constrasts, n = Inf) # Extract top-ranked genes

DEG <- na.omit(DEG) # Remove rows with missing values

fdr <- 0.05

k1 <- (DEG$adj.P.Val < fdr) & (DEG$logFC < (-1))

k2 <- (DEG$adj.P.Val < fdr) & (DEG$logFC > (1))

change <- ifelse(k1, "DOWN", ifelse(k2, "UP", "NOT"))

DEG$change <- change

nrow(DEG[DEG$change == "UP", ]) # High expression

nrow(DEG[DEG$change == "DOWN", ]) # Low expression

#----Save Data----

save(DEG, group, file = "dePMRG.Rdata")

#----Volcano Plot----

x_lim <- max(DEG$logFC, -DEG$logFC)

DEG$gene_name <- row.names(DEG)

DEG_sig <- DEG[abs(DEG$logFC) > 1 & DEG$adj.P.Val < 0.05, ]

pdf(file = "Volcano_Plot.pdf", height = 3, width = 3)

set.seed(56312)

colors <- sample(colors(), 2)

p <- ggplot(DEG, aes(logFC, -1 * log10(adj.P.Val), color = change)) +

geom_point(size = 2) +

theme_bw() +

xlim(-x_lim, x_lim) +

labs(x = "Log2(Fold Change)", y = "-Log10(P.adj)") +

scale_color_manual(values = c(colors[1], "grey", colors[2])) +

geom_hline(aes(yintercept = -1 * log10(0.05)), color = "black", linetype = "dashed") +

geom_vline(xintercept = c(-1, 1), color = "black", linetype = "dashed") +

theme(axis.text = element_text(size = 12),

axis.title = element_text(size = 12, face = "bold"),

legend.title = element_blank(),

legend.text = element_text(size = 10),

legend.position = "top") +

theme(panel.grid.major = element_blank(),

panel.grid.minor = element_blank()) +

theme(plot.title = element_text(hjust = 0.5, size = 12, face = "bold")) +

theme(legend.key.size = unit(0.5, "cm")) +

geom_text_repel(data = DEG_sig, aes(label = gene_name), size = 4)

print(p)

dev.off()

#------------------------Mutation Analysis------------------------

library(maftools)

load("TCGA-BRCA_MUTATIONS.rda")

mut_hrg <- all_mut[all_mut$Hugo_Symbol %in% geneset, ]

mut_hrg <- read.maf(mut_hrg)

# Generate oncoplot

oncoplot(maf = mut_hrg, fontSize = 0.6, showTumorSampleBarcodes = FALSE)

#--------------------------------Prognostic Analysis--------------------------------------

tumor_fpkm <- exp_fpkm_gene_name[, substr(colnames(exp_fpkm_gene_name), 14, 14) == "0"]

colnames(tumor_fpkm) <- substr(colnames(tumor_fpkm), 1, 12)

tumor_fpkm <- tumor_fpkm[, unique(colnames(tumor_fpkm))]

tumor_fpkm <- log2(tumor_fpkm + 1)

coxdata <- as.data.frame(t(tumor_fpkm))

coxdata <- coxdata[, colnames(coxdata) %in% rownames(DEG)]

coxdata$Patients <- rownames(coxdata)

coxdata <- merge(coxdata, tcga_clinical, by = "Patients", sort = FALSE)

# Single-factor Cox regression analysis

coxdata$status <- ifelse(coxdata$status == "Dead", 1, 0)

nrows <- nrow(DEG)

unicox_result <- data.frame(matrix(data = NA, nrow = nrows, ncol = 5))

for (i in 1:nrows) {

unicox <- coxph(Surv(days, status) ~ coxdata[, i + 1], data = coxdata)

unicox_result$X1[i] <- summary(unicox)[["conf.int"]][1]

unicox_result$X2[i] <- summary(unicox)[["conf.int"]][2]

unicox_result$X3[i] <- summary(unicox)[["conf.int"]][3]

unicox_result$X4[i] <- summary(unicox)[["conf.int"]][4]

unicox_result$X5[i] <- summary(unicox)[["coefficients"]][5]

}

rownames(unicox_result)[1:nrows] <- colnames(coxdata)[2:(nrows + 1)]

colnames(unicox_result) <- c("HR", "exp(-coef)", "Lower95", "Upper95", "p")

unicox_result_sig <- subset(unicox_result, p < 0.05)

unicox_result_sig$gene <- rownames(unicox_result_sig)

unicox_result_sig <- unicox_result_sig[order(unicox_result_sig$p, decreasing = TRUE), ]

unicox_result_sig$group <- ifelse(unicox_result_sig$HR > 1, "risk", "protective")

pro_genes <- rownames(unicox_result_sig) # prognostic PMRG = 46

write.csv(unicox_result_sig, file = "unicox_genes.csv")

# Plot error bar graph

df <- unicox_result

gene_name <- rownames(df)

df <- data.frame(df, gene_name, row.names = NULL)

df <- df[df$p < 0.05, ]

df <- df[df$HR < 10, ]

df <- df[order(df$HR), ]

# Convert p-values to log scale for color mapping

df$p_log10 <- -log10(df$p)

# Generate error bar plot using ggplot2

library(ggplot2)

ggplot(df, aes(x = gene_name, y = HR)) +

geom_point(size = 3) + # Points represent HR values

geom_errorbar(aes(ymin = Lower95, ymax = Upper95), width = 0.2) + # Error bars

geom_line(aes(group = gene_name), linetype = "dashed") + # Connect points

theme_bw() +

coord_flip() +

ylab("Hazard Ratio") +

xlab(NULL) +

geom_hline(yintercept = 1, linetype = "dashed", color = "black") # Add horizontal line

#-----------------------Clustering Analysis---------------------------------------

# Load required packages

library(ConsensusClusterPlus)

library(ggplot2)

library(reshape2)

library(survival)

library(survminer)

# Select cancer data for clustering analysis

data <- tumor_fpkm[rownames(tumor_fpkm) %in% pro_genes, ]

data <- as.matrix(data)

path <- getwd()

# Perform consensus clustering

results <- ConsensusClusterPlus(

data, # Columns are samples, rows are genes

maxK = 6, # Maximum number of clusters

reps = 50, # Number of resampling iterations

pItem = 0.8,

pFeature = 0.8,

seed = 12123,

clusterAlg = 'pam',

distance = 'pearson',

title = path,

plot = 'pdf'

)

# Calculate ICL (Integrated Classification Likelihood)

ic1 <- calcICL(results, title = path, plot = 'png')

# Determine the optimal number of clusters (K)

Kvec <- 2:6

x1 <- 0.1; x2 <- 0.9 # Thresholds defining the intermediate sub-interval

PAC <- rep(NA, length(Kvec))

names(PAC) <- paste("K=", Kvec, sep = "") # From 2 to maxK

for (i in Kvec) {

M <- results[[i]]$consensusMatrix

Fn <- ecdf(M[lower.tri(M)])

PAC[i - 1] <- Fn(x2) - Fn(x1) # Calculate PAC for each K

}

optK <- Kvec[which.min(PAC)] # Optimal number of clusters

# Compare the two largest clusters

clusters <- as.data.frame(results[[2]]["consensusClass"])

clusters$Patients <- rownames(clusters)

# Survival analysis

clinical <- tcga_clinical[, c("Patients", "status", "days")]

sadata <- merge(clusters, clinical, by = "Patients")

sadata$status <- ifelse(sadata$status == "Dead", 1, 0)

sadata$consensusClass <- ifelse(sadata$consensusClass == 1, "Cluster 1", "Cluster 2")

# Generate Kaplan-Meier plot

cluster_km <- survfit(Surv(days, status) ~ consensusClass, data = sadata)

pdf(file = "KM_clusters.pdf", height = 4, width = 4)

set.seed(56312)

colors <- sample(colors(), 2)

p <- ggsurvplot(

cluster_km,

size = 1,

linetype = "strata", # Change line type

palette = colors, # Custom color palette

pval = TRUE, # Add p-value

pval.method = TRUE,

surv.median.line = "hv",

conf.int = TRUE,

legend.title = "",

legend = c(0.7, 0.9),

legend.labs = c("Cluster 1", "Cluster 2"),

risk.table = FALSE

)

print(p)

dev.off()

#-----------------------PCA Analysis------------------------------

# Perform PCA on transcriptomic data

pca <- prcomp(as.matrix(t(data)), scale = TRUE)

# Convert PCA results to a data frame

pca_df <- as.data.frame(pca$x)

pcadata <- pca_df

pcadata$Patients <- rownames(pcadata)

pcadata <- merge(pcadata, clusters, by = "Patients", sort = FALSE)

pcadata$consensusClass <- ifelse(pcadata$consensusClass == 1, "Cluster 1", "Cluster 2")

pca_df$group <- pcadata$consensusClass

# Generate PCA plot

library(ggplot2)

pdf("pca.pdf", height = 3, width = 4)

pcaplot <- ggplot(pca_df, aes(x = PC1, y = PC2, color = group)) +

geom_point(size = 3) +

xlab(paste0("PC1 (", round(summary(pca)$importance[2, 1] * 100, 1), "%)")) +

ylab(paste0("PC2 (", round(summary(pca)$importance[2, 2] * 100, 1), "%)")) +

scale_color_manual(values = colors) +

theme_bw() +

theme(legend.position = "top")

print(pcaplot)

dev.off()

#-----------------------Prognostic Model Construction--------------------------------

library(glmnet)

# Prepare data for model construction

pro_genes <- intersect(pro_genes, rownames(geoexpr1)) # pro_genes = 37

x <- coxdata[, colnames(coxdata) %in% pro_genes]

x <- as.matrix(x)

y <- coxdata[, c("days", "status")]

names(y) <- c('time', 'status')

y$time <- as.double(y$time)

y$status <- as.double(y$status)

y <- as.matrix(survival::Surv(y$time, y$status))

# Train LASSO model

set.seed(12345)

fit <- glmnet(x, y, family = "cox")

pdf("lasso.lambda.pdf")

lambda <- plot(fit, xvar = "lambda", label = TRUE)

dev.off()

lasso_fit <- cv.glmnet(x, y, family = "cox", type.measure = 'deviance', maxit = 20000)

cvfit <- plot(lasso_fit)

cvfit + abline(v = log(c(lasso_fit$lambda.min, lasso_fit$lambda.1se)), lty = "dashed")

# Extract significant genes

coefficient <- coef(lasso_fit, s = lasso_fit$lambda.min)

active.index <- which(as.numeric(coefficient) != 0)

active.coefficients <- as.numeric(coefficient)[active.index]

sig_gene_multi_cox <- rownames(coefficient)[active.index]

length(sig_gene_multi_cox) # Number of significant genes

# Plot LASSO results

par(mfrow = c(1, 2))

plot(fit, xvar = "lambda", label = TRUE)

plot(lasso_fit)

cvfit + abline(v = log(c(lasso_fit$lambda.min, lasso_fit$lambda.1se)), lty = "dashed")

# Save significant genes

write.csv(sig_gene_multi_cox, file = "lasso_sig_genes_pattern.csv")

# Plot model coefficients

data <- data.frame(cof = active.coefficients, gene = sig_gene_multi_cox)

data$group <- ifelse(data$cof > 0, "Risk", "Protect")

set.seed(511)

colors <- sample(colors(), 2)

pdf(file = "model_coefficients.pdf", height = 3, width = 4)

p <- ggplot(data, aes(x = gene, y = cof, fill = group)) +

geom_bar(stat = "identity", position = "dodge") +

labs(x = "Gene", y = "Coefficient Value") +

geom_text(aes(label = format(cof, digits = 2)),

position = position_dodge(width = 0.9),

hjust = 0.1) +

theme_bw() +

scale_fill_manual(values = colors, name = "Group") +

coord_flip() +

xlab(NULL) +

theme(legend.position = "top")

print(p)

dev.off()

#-----------------------Model Construction and Evaluation------------------------------

# Calculate risk score

included_genes <- sig_gene_multi_cox

included_genes <- gsub("`", "", included_genes)

riskscore <- apply(coxdata[, included_genes], 1, function(k) {

sum(active.coefficients * k)

})

riskout <- cbind(coxdata, riskscore)

riskout$riskgroup <- ifelse(riskout$riskscore >= median(riskout$riskscore), "High", "Low")

# Generate TCGA cohort dot plot

set.seed(19238)

colors <- sample(colors(), 5)

fp_dat <- data.frame(patientsid = 1:length(riskscore), riskscore = as.numeric(sort(riskscore)))

fp_dat$riskgroup <- ifelse(fp_dat$riskscore >= median(fp_dat$riskscore), 'High', 'Low')

pdf(file = "TCGA_Cohort_Grouping.pdf", height = 3, width = 3)

p1 <- ggplot(fp_dat, aes(x = patientsid, y = riskscore)) +

geom_point(aes(color = riskgroup)) +

scale_color_manual(values = c(colors[2], colors[3])) +

theme_bw() + labs(x = "Patient ID", y = "Risk Score") +

geom_hline(yintercept = median(fp_dat$riskscore), color = "black", linetype = "dotted", linewidth = 0.8) +

geom_vline(xintercept = sum(fp_dat$riskgroup == "Low"), color = "black", linetype = "dotted", linewidth = 0.8) +

theme(legend.position = "top")

print(p1)

dev.off()

# Generate Kaplan-Meier curve

sur_fit <- survfit(Surv(days, status) ~ riskgroup, data = riskout)

pdf(file = "KM_Curve_TCGA.pdf", height = 3, width = 3)

train_km <- ggsurvplot(sur_fit,

size = 1,

linetype = 2, # Change line type

palette = c(colors[2], colors[3]), # Custom color palette

conf.int = TRUE, # Add confidence interval

pval = TRUE, # Add p-value

ggtheme = theme_bw()

)

print(train_km)

dev.off()

# Generate ROC curve

library(survivalROC)

roc1 <- survivalROC(Stime = riskout$days,

status = riskout$status,

marker = riskout$riskscore,

predict.time = 365 * 1,

method = "KM")

roc3 <- survivalROC(Stime = riskout$days,

status = riskout$status,

marker = riskout$riskscore,

predict.time = 365 * 3,

method = "KM")

roc5 <- survivalROC(Stime = riskout$days,

status = riskout$status,

marker = riskout$riskscore,

predict.time = 365 * 5,

method = "KM")

pdf(file = "ROC_Curve_TCGA.pdf", height = 3.5, width = 3.5)

plot(roc1$FP, roc1$TP,

type = "l", col = "red", xlim = c(0, 1), ylim = c(0, 1),

xlab = "False Positive Rate",

ylab = "True Positive Rate")

abline(0, 1, col = "gray", lty = 2)

lines(roc3$FP, roc3$TP, type = "l", col = "green",

xlim = c(0, 1), ylim = c(0, 1))

lines(roc5$FP, roc5$TP, type = "l", col = "blue",

xlim = c(0, 1), ylim = c(0, 1))

legend(0.2, 0.4, c(paste("1-y AUC=", round(roc1$AUC, 3)),

paste("3-y AUC=", round(roc3$AUC, 3)),

paste("5-y AUC=", round(roc5$AUC, 3))),

x.intersp = 1, y.intersp = 0.8,

lty = 1, lwd = 2, col = c("red", "green", "blue"),

bty = "n"

)

dev.off()

#-----------------------Validation Cohort Survival Analysis------------------------------

# Prepare test data

testdata <- as.data.frame(t(geoexpr1))

testdata <- log2(testdata + 1)

testdata <- testdata[, colnames(testdata) %in% included_genes]

testdata$riskscore <- apply(testdata[, included_genes], 1, function(k) {

sum(active.coefficients * k)

})

testdata$riskgroup <- ifelse(testdata$riskscore >= median(testdata$riskscore), "High", "Low")

testdata$title <- rownames(testdata)

colnames(geo_clinical)[1] <- "title"

testdata <- merge(testdata, geo_clinical, by = "title")

testdata$days <- as.numeric(testdata$days)

# Generate Kaplan-Meier curve for validation cohort

sur_fit_test <- survfit(Surv(days, status) ~ riskgroup, data = testdata)

pdf("Validation_KM.pdf", pointsize = 12, height = 3, width = 3)

test_km <- ggsurvplot(sur_fit_test,

size = 1,

linetype = "strata", # Change line type

palette = c(colors[2], colors[3]), # Custom color palette

conf.int = TRUE, # Add confidence interval

pval = TRUE, # Add p-value

ggtheme = theme_bw()

)

print(test_km)

dev.off()

# Generate GEO cohort dot plot

fp_dat <- data.frame(patientsid = 1:length(testdata$riskscore), riskscore = as.numeric(sort(testdata$riskscore)))

fp_dat$riskgroup <- ifelse(fp_dat$riskscore >= median(testdata$riskscore), 'High', 'Low')

pdf(file = "GEO_Cohort_Grouping.pdf", height = 3, width = 3)

p4 <- ggplot(fp_dat, aes(x = patientsid, y = riskscore)) +

geom_point(aes(color = riskgroup)) +

scale_color_manual(values = c(colors[2], colors[3])) +

theme_bw() + labs(x = "Patient ID", y = "Risk Score") +

geom_hline(yintercept = median(testdata$riskscore), color = "black", linetype = "dotted", linewidth = 0.8) +

geom_vline(xintercept = sum(fp_dat$riskgroup == "Low"), color = "black", linetype = "dotted", linewidth = 0.8) +

theme(legend.position = "top")

print(p4)

dev.off()

# Generate ROC curve for validation cohort

roc1 <- survivalROC(Stime = testdata$days,

status = testdata$status,

marker = testdata$riskscore,

predict.time = 365 * 1,

method = "KM")

roc3 <- survivalROC(Stime = testdata$days,

status = testdata$status,

marker = testdata$riskscore,

predict.time = 365 * 3,

method = "KM")

roc5 <- survivalROC(Stime = testdata$days,

status = testdata$status,

marker = testdata$riskscore,

predict.time = 365 * 5,

method = "KM")

pdf(file = "ROC_Curve_GEO.pdf", height = 3.5, width = 3.5)

plot(roc1$FP, roc1$TP,

type = "l", col = "red", xlim = c(0, 1), ylim = c(0, 1),

xlab = "False Positive Rate",

ylab = "True Positive Rate")

abline(0, 1, col = "gray", lty = 2)

lines(roc3$FP, roc3$TP, type = "l", col = "green",

xlim = c(0, 1), ylim = c(0, 1))

lines(roc5$FP, roc5$TP, type = "l", col = "blue",

xlim = c(0, 1), ylim = c(0, 1))

legend(0.2, 0.4, c(paste("1-y AUC=", round(roc1$AUC, 3)),

paste("3-y AUC=", round(roc3$AUC, 3)),

paste("5-y AUC=", round(roc5$AUC, 3))),

x.intersp = 1, y.intersp = 0.8,

lty = 1, lwd = 2, col = c("red", "green", "blue"),

bty = "n"

)

dev.off()

#----------------------Risk Score and Clinical Feature Correlation Analysis------------------------------

library(dplyr)

library(ComplexHeatmap)

# Prepare the dataset

n <- ncol(riskout)

data <- cbind(riskout[, c(1, (n - 10):n)], riskout[, sig_gene_multi_cox])

data$status <- ifelse(data$status == 0, "Alive", "Dead")

# Sort the data by risk group

data <- data[order(data$riskgroup), ]

# Prepare annotation data

annotation_df <- data[, c(2, 4:12)]

# Prepare heatmap data

numeric_data <- data[, 13:ncol(data)]

# Generate heatmap

library(circlize)

pdf(file = "Clinical_Feature_Heatmap.pdf", height = 6, width = 8)

set.seed(113)

colors <- sample(colors(), 3)

p <- pheatmap(

t(numeric_data),

color = colorRamp2(c(-2, 0, 2), c("blue", "white", "red")),

border_color = NA,

scale = "row",

cluster_rows = TRUE,

cluster_cols = FALSE,

legend = TRUE,

show_rownames = TRUE,

show_colnames = FALSE,

fontsize = 10,

annotation_col = annotation_df,

annotation_legend = TRUE,

annotation_names_col = TRUE

)

print(p)

dev.off()

# Generate boxplots for risk score vs. status and chemotherapy

P1 <- ggplot(data, aes(x = status, y = riskscore)) +

labs(y = "Risk Score", x = "Status", title = NULL) +

geom_violin(aes(fill = status), position = position_dodge(0.5), alpha = 0.25) +

geom_boxplot(

aes(fill = status),

position = position_dodge(0.5),

outlier.shape = 20,

alpha = 0.5

) +

scale_fill_manual(values = c(colors[1], colors[2])) +

theme_classic() +

stat_compare_means(

comparisons = list(c("Alive", "Dead")),

label = "p.signif",

method = "wilcox.test",

hide.ns = FALSE,

label.x.npc = "center"

) + theme(legend.position = "none")

P2 <- ggplot(data, aes(x = chemotherapy, y = riskscore)) +

labs(y = "Risk Score", x = "Chemotherapy", title = NULL) +

geom_violin(aes(fill = chemotherapy), position = position_dodge(0.5), alpha = 0.25) +

geom_boxplot(

aes(fill = chemotherapy),

position = position_dodge(0.5),

outlier.shape = 20,

alpha = 0.5

) +

scale_fill_manual(values = c(colors[1], colors[2], colors[3], colors[4])) +

theme_classic() +

stat_compare_means(

comparisons = list(c("YES", "NO")),

label = "p.signif",

method = "wilcox.test",

hide.ns = FALSE,

label.x.npc = "center"

) + theme(legend.position = "none")

# Combine plots

library(cowplot)

combined_plot <- plot_grid(P1, P2, nrow = 2, ncol = 1)

# Save the combined plot

ggsave(

"Clinical_Correlation.pdf",

combined_plot,

width = 5,

height = 10,

units = "cm"

)

#----------------------Mutation Analysis-----------------------------------

library(maftools)

load("TCGA-BRCA_MUTATIONS.rda")

tmb <- read.csv("tmb_results.csv", header = TRUE)

all_mut$Tumor_Sample_Barcode <- substr(all_mut$Tumor_Sample_Barcode, 1, 12)

all_mut <- all_mut[all_mut$Tumor_Sample_Barcode %in% riskout$Patients, ]

all_mut1 <- read.maf(all_mut)

all_mut1@clinical.data$group <- riskout$riskgroup[match(all_mut1@clinical.data$Tumor_Sample_Barcode, riskout$Patients)]

# Generate oncoplot

oncoplot(all_mut1, top = 10, clinicalFeatures = "group", sortByAnnotation = TRUE)

# Merge TMB data

tmb$Tumor_Sample_Barcode <- substr(tmb$Tumor_Sample_Barcode, 1, 12)

colnames(tmb)[2] <- "Patients"

data <- merge(data, tmb, by = "Patients", sort = FALSE)

# Generate TMB boxplot

p <- ggplot(data, aes(x = riskgroup, y = total_perMB)) +

labs(y = "TMB", x = NULL, title = NULL) +

geom_violin(aes(fill = riskgroup), position = position_dodge(0.5), alpha = 0.25) +

geom_boxplot(

aes(fill = riskgroup),

position = position_dodge(0.5),

outlier.shape = 20,

alpha = 0.5

) +

scale_fill_manual(values = c(colors[1], colors[2], colors[3], colors[4])) +

theme_classic() +

stat_compare_means(

comparisons = list(c("high", "low")),

label = "p.signif",

method = "wilcox.test",

hide.ns = FALSE,

label.x.npc = "center"

) + theme(legend.position = "none")

ggsave(

"TMB_Group.pdf",

p,

width = 5,

height = 5,

units = "cm"

)

# Calculate correlation coefficient and p-value

correlation_coefficient <- round(cor(data$riskscore, data$total_perMB, method = "pearson"), 2)

p_value <- format.pval(cor.test(data$riskscore, data$total_perMB, method = "pearson")$p.value, digits = 2)

# Create scatter plot with marginal density plots

p <- ggplot(data, aes(x = riskscore, y = total_perMB)) +

geom_point() +

stat_smooth(method = lm, se = FALSE) +

labs(x = "Risk Scores", y = "Tumor Mutation Burden") +

theme_bw() +

theme(legend.position = "none")

# Add correlation coefficient and p-value

p <- p + annotate(

"text",

x = 0.51,

y = 0.35,

label = paste0("r = ", correlation_coefficient, ",\nP = ", p_value),

parse = FALSE,

hjust = 0,

vjust = 0,

size = 4,

color = "red"

)

# Add marginal density plots

library(ggExtra)

p_with_marginals <- ggExtra::ggMarginal(

p,

type = "density",

xparams = list(fill = "green"),

yparams = list(fill = "orange")

)

ggsave(

"TMB_RiskScore.pdf",

p_with_marginals,

width = 5,

height = 5,

units = "cm"

)

#----------------------Drug Sensitivity Analysis------------------------------

library(pRRophetic)

library(sva)

library(car)

library(genefilter)

library(preprocessCore)

library(ridge)

library(ggplot2)

library(ggpubr)

library(survminer)

library(reshape2)

library(tidyr)

# Define drugs for analysis

all_drugs <- c(

"Axitinib", "Bexarotene", "Bicalutamide", "Bleomycin", "Bortezomib", "Bosutinib", "Camptothecin", "Cisplatin", "Cyclopamine",

"Cytarabine", "Dasatinib", "Docetaxel", "Doxorubicin", "Elesclomol", "Embelin", "Epothilone.B", "Erlotinib", "Etoposide",

"Gefitinib", "Gemcitabine", "Imatinib", "Lapatinib", "Lenalidomide", "Metformin", "Methotrexate", "Midostaurin", "Nilotinib",

"Obatoclax.Mesylate", "Paclitaxel", "Parthenolide", "Pazopanib", "Pyrimethamine", "Rapamycin", "Roscovitine", "Salubrinal",

"Shikonin", "Sorafenib", "S.Trityl.L.cysteine", "Sunitinib", "Temsirolimus", "Thapsigargin", "Tipifarnib", "Vinblastine",

"Vinorelbine", "Vorinostat"

)

# Load necessary scripts

source("compute_phenotype_function.R")

source("predict_from_cgp.R")

source("classification_function.R")

source("do_variable_selection.R")

source("homogenize_data.R")

source("summarizeGenesByMean.R")

# Prepare expression data

chedata <- tumor_fpkm[, colnames(tumor_fpkm) %in% riskout$Patients]

# Define groups

groups <- data.frame(patients = colnames(chedata), no = c(1:ncol(chedata)))

cluster <- data.frame(patients = riskout$Patients, group = riskout$riskgroup)

groups <- merge(groups, cluster, by = "patients", sort = FALSE)

group <- groups$group

table(group)

# Process data and predict drug sensitivities

all_zero_genes <- apply(chedata, 1, function(x) all(x == 0))

chedata <- chedata[!all_zero_genes, ]

chedata <- as.matrix(chedata)

# Loop through each drug to predict sensitivity

l <- length(all_drugs)

p <- ncol(chedata)

results <- data.frame()

for (i in 1:l) {

predictedPtype <- pRRopheticPredict(

testMatrix = chedata,

drug = all_drugs[i],

tissueType = "all",

batchCorrect = "eb",

selection = 1

)

df <- as.data.frame(cbind(predictedPtype, group))

df$drug <- rep(all_drugs[i], p)

results <- rbind(results, df)

}

results$predictedPtype <- as.numeric(results$predictedPtype)

colnames(results)[1] <- "value"

results1 <- results[!is.na(results$value), ]

save(results1, file = "Drug_Sensitivity_Analysis.rda")

# Compare drug sensitivities between risk groups

load("Drug_Sensitivity_Analysis.rda")

set.seed(723)

colors <- sample(colors(), 2)

pdf(file = "Drug_Sensitivity_Group.pdf", height = 5, width = 10)

p <- ggplot(results1, aes(x = drug, y = value)) +

labs(y = "Predicted Sensitivity", x = NULL, title = NULL) +

geom_boxplot(aes(fill = group)) +

scale_fill_manual(values = colors) +

theme_classic() +

stat_compare_means(

aes(group = group),

label = "p.signif",

method = "wilcox.test",

hide.ns = TRUE

) +

theme(axis.text.x = element_text(angle = -90, hjust = 0.1)) +

theme(legend.position = "top")

print(p)

dev.off()

# Correlation analysis between drug sensitivity, risk score, and prognostic gene expression

results1$patients <- substr(rownames(results1), 1, 12)

drug_data <- pivot_wider(

results1,

id_cols = "patients",

names_from = "drug",

values_from = "value"

)

data <- riskout[, c("Patients", "riskscore", sig_gene_multi_cox)]

colnames(drug_data)[1] <- "Patients"

drug_data <- merge(drug_data, data, by = "Patients", sort = FALSE)

# Generate correlation heatmap

library(corrplot)

drug_data <- drug_data[, -1]

corr_drug <- cor(drug_data)

corr_drug <- corr_drug[1:45, 46:63]

corr_drug <- t(corr_drug)

pdf("Drug_Sensitivity_Correlation.pdf", height = 5, width = 15)

p2 <- corrplot(

corr_drug,

method = "square",

type = "full",

tl.col = "black",

tl.cex = 0.8,

order = "hclust",

hclust.method = "complete"

)

print(p2)

dev.off()

#------------------Immune Infiltration Analysis------------------------------

# Load required packages

library(IOBR)

library(ggplot2)

library(cowplot)

library(reshape2)

# Load data

data <- riskout[, c("Patients", "riskgroup", "riskscore")]

# Perform immune infiltration analysis

immudata <- tumor_fpkm[, colnames(tumor_fpkm) %in% riskout$Patients]

# Use different deconvolution methods to estimate immune cell composition

cibersort <- deconvo_tme(eset = immudata, method = "cibersort", arrays = TRUE, perm = 100)

epic <- deconvo_tme(eset = immudata, method = "epic", arrays = TRUE)

xcell <- deconvo_tme(eset = immudata, method = "xcell", arrays = TRUE)

mcp <- deconvo_tme(eset = immudata, method = "mcpcounter")

estimate <- deconvo_tme(eset = immudata, method = "estimate")

timer <- deconvo_tme(eset = immudata, method = "timer", group_list = rep("stad", dim(immudata)[2]))

quantiseq <- deconvo_tme(eset = immudata, tumor = TRUE, arrays = TRUE, scale_mrna = TRUE, method = "quantiseq")

ips <- deconvo_tme(eset = immudata, method = "ips", plot = FALSE)

# Merge results from different methods

innune_inf <- merge(cibersort, epic, by = "ID", sort = FALSE)

innune_inf <- merge(innune_inf, xcell, by = "ID", sort = FALSE)

innune_inf <- merge(innune_inf, mcp, by = "ID", sort = FALSE)

innune_inf <- merge(innune_inf, timer, by = "ID", sort = FALSE)

innune_inf <- merge(innune_inf, quantiseq, by = "ID", sort = FALSE)

colnames(innune_inf)[1] <- "Patients"

innune_inf$group <- data$riskgroup

innune_inf$riskscore <- data$riskscore

innune_inf <- innune_inf[order(innune_inf$group), ]

innune_inf <- innune_inf %>% select_if(~!all(.x == 0))

# Save the results

save(cibersort, epic, xcell, mcp, estimate, timer, quantiseq, ips, file = "免疫浸润分析结果.rda")

#------------------Group Comparison and Visualization------------------------

load("免疫浸润分析结果.rda")

set.seed(569)

colors <- sample(colors(), 2)

df <- cibersort[, 1:23]

colnames(df)[1] <- "Patients"

df$group <- riskout$riskgroup[match(df$Patients, riskout$Patients)]

df1 <- melt(df)

df$riskscore <- riskout$riskscore[match(df$Patients, riskout$Patients)]

df <- df[order(df$group), ]

df1$variable <- gsub("(.*)_CIBERSORT$", "\\1", df1$variable)

# Plot cell composition differences

ggplot(df1, aes(x = variable, y = value)) +

labs(y = "Cell Composition", x = NULL, title = NULL) +

geom_boxplot(aes(fill = group), position = position_dodge(0.5)) +

scale_fill_manual(values = colors) +

theme_classic() +

stat_compare_means(

aes(group = group),

label = "p.signif",

method = "wilcox.test",

hide.ns = FALSE

) +

theme(axis.text.x = element_text(angle = 45, hjust = 1)) +

theme(legend.position = "top")

#------------------ESTIMATE/IPA Scores Visualization-------------------------

colnames(ips)[1] <- "Patients"

colnames(estimate)[1] <- "Patients"

data <- riskout[, c("Patients", "riskscore", "riskgroup")]

data1 <- merge(estimate, data, by = "Patients", sort = FALSE)

data1 <- merge(ips, data1, by = "Patients", sort = FALSE)

set.seed(569)

colors <- sample(colors(), 2)

# Plot ESTIMATE scores

P1 <- ggplot(data1, aes(x = riskgroup, y = StromalScore_estimate)) +

labs(y = "Stromal Score", x = "Risk Group", title = NULL) +

geom_violin(aes(fill = riskgroup), position = position_dodge(0.5), alpha = 0.25) +

geom_boxplot(

aes(fill = riskgroup),

position = position_dodge(0.5),

outlier.shape = 20,

alpha = 0.5

) +

scale_fill_manual(values = colors) +

theme_classic() +

stat_compare_means(

comparisons = list(c("high", "low")),

label = "p.signif",

method = "wilcox.test",

hide.ns = FALSE,

label.x.npc = "center"

) + theme(legend.position = "none")

P2 <- ggplot(data1, aes(x = riskgroup, y = ImmuneScore_estimate)) +

labs(y = "Immune Score", x = "Risk Group", title = NULL) +

geom_violin(aes(fill = riskgroup), position = position_dodge(0.5), alpha = 0.25) +

geom_boxplot(

aes(fill = riskgroup),

position = position_dodge(0.5),

outlier.shape = 20,

alpha = 0.5

) +

scale_fill_manual(values = colors) +

theme_classic() +

stat_compare_means(

comparisons = list(c("high", "low")),

label = "p.signif",

method = "wilcox.test",

hide.ns = FALSE,

label.x.npc = "center"

) + theme(legend.position = "none")

P3 <- ggplot(data1, aes(x = riskgroup, y = ESTIMATEScore_estimate)) +

labs(y = "ESTIMATE Score", x = "Risk Group", title = NULL) +

geom_violin(aes(fill = riskgroup), position = position_dodge(0.5), alpha = 0.25) +

geom_boxplot(

aes(fill = riskgroup),

position = position_dodge(0.5),

outlier.shape = 20,

alpha = 0.5

) +

scale_fill_manual(values = colors) +

theme_classic() +

stat_compare_means(

comparisons = list(c("high", "low")),

label = "p.signif",

method = "wilcox.test",

hide.ns = FALSE,

label.x.npc = "center"

) + theme(legend.position = "none")

P4 <- ggplot(data1, aes(x = riskgroup, y = TumorPurity_estimate)) +

labs(y = "Tumor Purity", x = "Risk Group", title = NULL) +

geom_violin(aes(fill = riskgroup), position = position_dodge(0.5), alpha = 0.25) +

geom_boxplot(

aes(fill = riskgroup),

position = position_dodge(0.5),

outlier.shape = 20,

alpha = 0.5

) +

scale_fill_manual(values = colors) +

theme_classic() +

stat_compare_means(

comparisons = list(c("high", "low")),

label = "p.signif",

method = "wilcox.test",

hide.ns = FALSE,

label.x.npc = "center"

) + theme(legend.position = "none")

P5 <- ggplot(data1, aes(x = riskgroup, y = AZ_IPS)) +

labs(y = "AZ IPS", x = "Risk Group", title = NULL) +

geom_violin(aes(fill = riskgroup), position = position_dodge(0.5), alpha = 0.25) +

geom_boxplot(

aes(fill = riskgroup),

position = position_dodge(0.5),

outlier.shape = 20,

alpha = 0.5

) +

scale_fill_manual(values = colors) +

theme_classic() +

stat_compare_means(

comparisons = list(c("high", "low")),

label = "p.signif",

method = "wilcox.test",

hide.ns = FALSE,

label.x.npc = "center"

) + theme(legend.position = "none")

#------------------Correlation Heatmap-------------------------

df <- cibersort[, 1:23]

colnames(df) <- gsub("(.*)_CIBERSORT$", "\\1", colnames(df))

colnames(df)[1] <- "Patients"

expdata <- riskout[, c("Patients", "riskscore", sig_gene_multi_cox)]

df1 <- merge(df, expdata, by = "Patients", sort = FALSE)

library(corrplot)

df1 <- df1[, -1]

corr_drug <- cor(df1)

corr_drug <- corr_drug[1:22, 23:40]

corr_drug <- t(corr_drug)

corrplot(

corr_drug,

method = "color",

type = "full",

tl.col = "black",

rect.lwd = 2,

tl.cex = 0.7

)

#------------------TIDE Analysis-------------------------

average <- mean(as.matrix(tumor_fpkm))

highp <- riskout[riskout$riskgroup == "high", ]$Patients

lowp <- riskout[riskout$riskgroup == "low", ]$Patients

fpc_high <- as.matrix(tumor_fpkm[, colnames(tumor_fpkm) %in% highp])

fpc_low <- as.matrix(tumor_fpkm[, colnames(tumor_fpkm) %in% lowp])

high_tide <- fpc_high - average

low_tide <- fpc_low - average

write.table(high_tide, file = "high_tide.txt", sep = "\t")

write.table(low_tide, file = "low_tide.txt", sep = "\t")

#------------------TIDE Results Visualization-------------------------

tide_high <- read.csv("tide_high.csv", header = TRUE)

tide_low <- read.csv("low_tide.csv", header = TRUE)

tide_high$group <- rep("high", nrow(tide_high))

tide_low$group <- rep("low", nrow(tide_low))

tide_data <- rbind(tide_high, tide_low)

# Plot TIDE results

P6 <- ggplot(tide_data, aes(x = group, y = TIDE)) +

labs(y = "TIDE", x = NULL, title = NULL) +

geom_violin(aes(fill = group), position = position_dodge(0.5), alpha = 0.25) +

geom_boxplot(

aes(fill = group),

position = position_dodge(0.5),

outlier.shape = 20,

alpha = 0.5

) +

scale_fill_manual(values = colors) +

theme_classic() +

stat_compare_means(

comparisons = list(c("high", "low")),

label = "p.signif",

method = "wilcox.test",

hide.ns = FALSE,

label.x.npc = "center"

) + theme(legend.position = "none")

# Combine all plots

combined_plot <- plot_grid(P1, P2, P3, P4, P5, P6, nrow = 2, ncol = 3)

# Save the combined plot as a PDF file

ggsave(

"ESTIMATE_tide.pdf",

combined_plot,

width = 20,

height = 12,

units = "cm"

)

#----------------------------nomogram-------------------------------

# Load required packages

library(rms)

library(survival)

library(survminer)

library(rmda)

library(stringr)

# Prepare the dataset for nomogram by integrating risk scores and clinical data

nomodata <- data.frame(Patients = riskout$Patients, riskscore = riskout$riskscore)

nomodata <- merge(nomodata, tcga_clinical, by = "Patients", sort = FALSE)

nomodata$status <- ifelse(nomodata$status == "Dead", 1, 0) # Convert status to binary

nomodata$N <- ifelse(nomodata$N == "NX", "N3", nomodata$N) # Handle missing N stage

save(nomodata, file = "nomodata.rda")

# Convert categorical variables to numerical values

nomodata$age <- ifelse(nomodata$age == ">=60", 0, 1)

nomodata$chemotherapy <- ifelse(nomodata$chemotherapy == "YES", 0, 1)

nomodata$radiotherapy <- ifelse(nomodata$radiotherapy == "YES", 0, 1)

nomodata$stage <- ifelse(nomodata$stage == "I", 0,

ifelse(nomodata$stage == "II", 1,

ifelse(nomodata$stage == "III", 2, 3)))

nomodata$T <- ifelse(nomodata$T == "T1", 0,

ifelse(nomodata$T == "T2", 1,

ifelse(nomodata$T == "T3", 2, 3)))

nomodata$N <- ifelse(nomodata$N == "N0", 0,

ifelse(nomodata$N == "N1", 1,

ifelse(nomodata$N == "N2", 2, 3)))

nomodata$M <- ifelse(nomodata$M == "M0", 0,

ifelse(nomodata$M == "M1", 1, 2))

# Save the processed dataset

write.csv(nomodata, file = "nomodata.csv")

#---------------------------Univariate Cox Regression---------------------------

# Define variables for univariate Cox regression

covariates <- c("riskscore", "age", "chemotherapy", "radiotherapy", "stage", "T", "N", "M")

# Create formulas for each variable

univ_formulas <- sapply(covariates,

function(x) as.formula(paste('Surv(days, status) ~', x)))

# Perform univariate Cox regression for each variable

univ_models <- lapply(univ_formulas, function(x) coxph(x, data = nomodata))

# Extract HR, 95% CI, and p-value

univ_results <- lapply(univ_models,

function(x) {

x <- summary(x)

p.value <- signif(x$wald["pvalue"], digits = 2)

HR <- signif(exp(x$coef), digits = 2)

HR.confint.lower <- signif(x$conf.int[,"lower .95"], 2)

HR.confint.upper <- signif(x$conf.int[,"upper .95"], 2)

HR1 <- paste0(HR, " [", HR.confint.lower, "-", HR.confint.upper, "]")

res <- c(p.value, HR, HR.confint.lower, HR.confint.upper, HR1)

names(res) <- c("p.value", "HR (95% CI for HR)")

return(res)

})

# Convert results to a dataframe

res <- t(as.data.frame(univ_results, check.names = FALSE))

res <- data.frame(rownames(res), res)

colnames(res) <- c("Characteristics", "HR", "lower.95", "upper.95", "HR[cofint]", "p")

res$HR <- as.numeric(res$HR)

res$lower.95 <- as.numeric(res$lower.95)

res$upper.95 <- as.numeric(res$upper.95)

# Generate forest plot

forestplot(

res[, c(1, 5, 6)],

mean = res[, 2],

lower = res[, 3],

upper = res[, 4],

zero = 1,

boxsize = 0.6,

graph.pos = "right",

hrzl_lines = list("1" = gpar(lty = 1, lwd = 2),

"2" = gpar(lty = 2),

"10" = gpar(lwd = 2, lty = 1, columns = c(1:4))),

graphwidth = unit(0.25, "npc"),

xlab = "Hazard ratio",

xticks = c(0.4, 1, 3, 5, 7, 10),

is.summary = c(T, F, F, F, F, F, F, F, F),

txt_gp = fpTxtGp(

label = gpar(cex = 1),

ticks = gpar(cex = 1),

xlab = gpar(cex = 1.5),

title = gpar(cex = 2)),

lwd.zero = 1,

lwd.ci = 1.5,

lwd.xaxis = 2,

lty.ci = 1.5,

ci.vertices = TRUE,

ci.vertices.height = 0.2,

clip = c(0.1, 8),

ineheight = unit(8, 'mm'),

line.margin = unit(8, 'mm'),

colgap = unit(6, 'mm'),

fn.ci_norm = "fpDrawDiamondCI",

col = fpColors(box = "blue", lines = "blue", zero = "black")

)

#---------------------------Multivariate Cox Regression---------------------------

# Perform multivariate Cox regression

mul_cox <- coxph(Surv(days, status) ~ riskscore + age + chemotherapy + radiotherapy +

stage + T + N + M, data = nomodata)

# Extract HR, 95% CI, and p-value

mul_cox1 <- summary(mul_cox)

multi1 <- as.data.frame(round(mul_cox1$conf.int[, c(1, 3, 4)], 2))

multi2 <- ShowRegTable(mul_cox, exp = TRUE, digits = 2, pDigits = 6,

printToggle = TRUE, quote = FALSE, ciFun = confint)

# Combine results into a dataframe

result <- cbind(multi1, multi2)

result <- tibble::rownames_to_column(result, var = "Characteristics")

colnames(result)[5] <- "HR[confint]"

result <- rbind(colnames(result), result)

# Generate forest plot

forestplot(

result[, c(1, 5, 6)],

mean = result[, 2],

lower = result[, 3],

upper = result[, 4],

zero = 1,

boxsize = 0.6,

graph.pos = "right",

hrzl_lines = list("1" = gpar(lty = 1, lwd = 2),

"2" = gpar(lty = 2),

"10" = gpar(lwd = 2, lty = 1, columns = c(1:4))),

graphwidth = unit(0.25, "npc"),

xlab = "Hazard ratio",

xticks = c(0.4, 1, 3, 5, 7, 10),

is.summary = c(T, F, F, F, F, F, F, F, F),

txt_gp = fpTxtGp(

label = gpar(cex = 1),

ticks = gpar(cex = 1),

xlab = gpar(cex = 1.5),

title = gpar(cex = 2)),

lwd.zero = 1,

lwd.ci = 1.5,

lwd.xaxis = 2,

lty.ci = 1.5,

ci.vertices = TRUE,

ci.vertices.height = 0.2,

clip = c(0.1, 8),

ineheight = unit(8, 'mm'),

line.margin = unit(8, 'mm'),

colgap = unit(6, 'mm'),

fn.ci_norm = "fpDrawDiamondCI",

col = fpColors(box = "blue", lines = "blue", zero = "black")

)

#---------------------------Nomogram Construction and Validation---------------------------

# Load the processed dataset

load("nomodata.rda")

# Prepare data for nomogram

ddist <- datadist(nomodata)

options(datadist = 'ddist')

# Build the multivariate Cox regression model

f <- cph(Surv(days, status) ~ riskscore + age + radiotherapy + chemotherapy + stage,

x = TRUE, y = TRUE, surv = TRUE,

data = nomodata, time.inc = 365 * 1)

# Calculate survival probabilities

surv <- Survival(f)

surv1 <- function(x) surv(365 * 1, lp = x)

surv3 <- function(x) surv(365 * 3, lp = x)

surv5 <- function(x) surv(365 * 5, lp = x)

# Build and visualize the nomogram

nomo <- nomogram(f,

fun = list(surv1, surv3, surv5),

funlabel = c("1-year survival", "3-year survival", "5-year survival"),

lp = FALSE,

maxscale = 100,

fun.at = c(0.99, 0.9, 0.7, 0.5, 0.3, 0.1))

plot(nomo)

# Validate the nomogram using bootstrapping

v <- validate(f, method = "boot", B = 1000, dxy = TRUE)

Dxy <- v[rownames(v) == "Dxy", colnames(v) == "index.corrected"]

orig_Dxy <- v[rownames(v) == "Dxy", colnames(v) == "index.orig"]

bias_corrected_c_index <- abs(Dxy) / 2 + 0.5

orig_c_index <- abs(orig_Dxy) / 2 + 0.5

# Calculate c-index and 95% CI

c_index <- rcorrcens(Surv(nomodata$days, nomodata$status) ~ predict(f))

c_index <- (1 - c_index[1])

# Generate calibration curves

cal <- calibrate(f, cmethod = 'KM', method = "boot", u = 365 * 1, m = 250, B = 1000)

cal3 <- calibrate(f3, cmethod = 'KM', method = "boot", u = 365 * 3, m = 250, B = 1000)

cal5 <- calibrate(f5, cmethod = 'KM', method = "boot", u = 365 * 5, m = 250, B = 1000)

# Plot calibration curves

pdf("calibration_curve.pdf")

set.seed(541)

colors <- sample(colors(), 3)

plot(cal, lwd = 2, lty = 1, conf.int = TRUE, errbar.col = colors[1], col = colors[1],

xlim = c(0.5, 1), ylim = c(0.5, 1), xlab = "Nomogram-predicted OS (%)",

ylab = "Observed OS (%)", subtitle = FALSE)

plot(cal3, lwd = 2, lty = 1, conf.int = TRUE, errbar.col = colors[2], col = colors[2],

xlim = c(0.5, 1), ylim = c(0.5, 1), subtitle = FALSE, add = TRUE)

plot(cal5, lwd = 2, lty = 1, conf.int = TRUE, errbar.col = colors[3], col = colors[3],

xlim = c(0.5, 1), ylim = c(0.5, 1), subtitle = FALSE, add = TRUE)

legend("topleft", legend = c("1-year", "3-year", "5-year"), col = colors,

lwd = 2, cex = 1.2, bty = "n")

dev.off()

#---------------------------Decision Curve Analysis (DCA)---------------------------

set.seed(176)

dca_nomogram <- decision_curve(status ~ riskscore + chemotherapy + stage + N, data = nomodata,

thresholds = seq(0, 1, by = 0.01), study.design = 'cohort',

bootstraps = 1000)

dca_chemotherapy <- decision_curve(status ~ chemotherapy, data = nomodata,

thresholds = seq(0, 1, by = 0.01), study.design = 'cohort',

bootstraps = 1000)

dca_radiotherapy <- decision_curve(status ~ radiotherapy, data = nomodata,

thresholds = seq(0, 1, by = 0.01), study.design = 'cohort',

bootstraps = 1000)

dca_age <- decision_curve(status ~ age, data = nomodata,

thresholds = seq(0, 1, by = 0.01), study.design = 'cohort',

bootstraps = 1000)

dca_stage <- decision_curve(status ~ stage, data = nomodata,

thresholds = seq(0, 1, by = 0.01), study.design = 'cohort',

bootstraps = 1000)

dca_riskscore <- decision_curve(status ~ riskscore, data = nomodata,

thresholds = seq(0, 1, by = 0.01), study.design = 'cohort',

bootstraps = 1000)

# Plot DCA

pdf("DCA.pdf", height = 4, width = 4)

plot_decision_curve(list(dca_nomogram, dca_riskscore, dca_chemotherapy, dca_radiotherapy,

dca_age, dca_stage),

curve.names = c("nomogram", "riskscore", "chemotherapy", "radiotherapy", "age", "stage"),

col = colors, lty = c(1, 1, 1, 1, 1, 1), lwd = c(2, 2, 2, 2, 2, 2),

legend.position = "topright", confidence.intervals = FALSE,

cost.benefit.axis = FALSE, xlim = c(0, 1))

dev.off()

#---------------------------ROC Curve---------------------------

library(survivalROC)

total_survival <- predict(f, newdata = nomodata)

nobs <- NROW(nomodata)

cutoff <- 365

roc1 <- survivalROC(Stime = nomodata$days, status = nomodata$status, marker = total_survival,

predict.time = cutoff, span = 0.25 * nobs^(-0.20))

roc2 <- survivalROC(Stime = nomodata$days, status = nomodata$status, marker = total_survival,

predict.time = 3 * cutoff, span = 0.25 * nobs^(-0.20))

roc3 <- survivalROC(Stime = nomodata$days, status = nomodata$status, marker = total_survival,

predict.time = 5 * cutoff, span = 0.25 * nobs^(-0.20))

# Plot ROC curves

pdf(file = "roc_curve_nomo.pdf", height = 4.5, width = 4)

set.seed(11)

colors <- sample(colors(), 3)

plot(roc1$FP, roc1$TP, type = "l", col = colors[1], xlim = c(0, 1), ylim = c(0, 1),

xlab = "False positive rate", ylab = "True positive rate")

abline(0, 1, col = "gray", lty = 2)

lines(roc2$FP, roc2$TP, type = "l", col = colors[2], xlim = c(0, 1), ylim = c(0, 1))

lines(roc3$FP, roc3$TP, type = "l", col = colors[3], xlim = c(0, 1), ylim = c(0, 1))

legend(0.4, 0.2, c(paste("AUC of 1 year=", round(roc1$AUC, 3)),

paste("AUC of 3 year=", round(roc2$AUC, 3)),

paste("AUC of 5 year=", round(roc3$AUC, 3))),

x.intersp = 1, y.intersp = 0.8, lty = 1, lwd = 2, col = colors,

bty = "n", seg.len = 1, cex = 0.8)

dev.off()

#-------------------------Prognostic Gene Expression-------------------------------

library(pheatmap)

data <- read.csv(file = "Expression_Public_23Q4_subsetted (1).csv", header = TRUE)

# Generate a heatmap

# First, sort the annotation data by riskgroup

annotation_df <- data[, c("lineage_2", "lineage_3")]

rownames(annotation_df) <- data$cell_line_display_name

# Then, extract and arrange the numeric variables in the same order for generating the heatmap

numeric_data <- t(data[, 9:13])

colnames(numeric_data) <- data$cell_line_display_name

# Finally, generate the heatmap using the sorted numeric data and annotation information

pheatmap(numeric_data,

color = colorRampPalette(c('blue', 'white', 'red'))(200),

border_color = "black",

scale = "row",

cluster_rows = FALSE,

cluster_cols = TRUE,

legend = TRUE,

show_rownames = TRUE,

show_colnames = TRUE,

fontsize = 8,

annotation_col = annotation_df,

annotation_legend = TRUE,

annotation_names_col = TRUE

)

# Gene expression in TCGA-BRCA and GEO datasets

data <- exp_fpkm_gene_name[rownames(exp_fpkm_gene_name) %in% sig_gene_multi_cox, ]

data <- as.data.frame(t(data))

data <- log2(data + 1)

data$group <- ifelse(substr(rownames(data), 14, 14) == "0",

"tumor",

"normal")

table(data$group)

set.seed(431)

colors <- sample(colors(), 2)

P1 <- ggplot(data, aes(x = group, y = TUBA1C)) +

labs(y = "gene expression level", x = NULL, title = NULL) +

geom_violin(aes(fill = group), position = position_dodge(0.5), alpha = 0.25) +

geom_boxplot(

aes(fill = group),

position = position_dodge(0.5),

outlier.shape = 20,

alpha = 0.5

) +

scale_fill_manual(values = colors) +

theme_classic() +

stat_compare_means(

comparisons = list(c("tumor", "normal")),

label = "p.signif",

method = "wilcox.test",

hide.ns = FALSE,

label.x.npc = "center"

) + theme(legend.position = "none")

P2 <- ggplot(data, aes(x = group, y = TUBA3D)) +

labs(y = "gene expression level", x = NULL, title = NULL) +

geom_violin(aes(fill = group), position = position_dodge(0.5), alpha = 0.25) +

geom_boxplot(

aes(fill = group),

position = position_dodge(0.5),

outlier.shape = 20,

alpha = 0.5

) +

scale_fill_manual(values = colors) +

theme_classic() +

stat_compare_means(

comparisons = list(c("tumor", "normal")),

label = "p.signif",

method = "wilcox.test",

hide.ns = FALSE,

label.x.npc = "center"

) + theme(legend.position = "none")

P3 <- ggplot(data, aes(x = group, y = TUBA3E)) +

labs(y = "gene expression level", x = NULL, title = NULL) +

geom_violin(aes(fill = group), position = position_dodge(0.5), alpha = 0.25) +

geom_boxplot(

aes(fill = group),

position = position_dodge(0.5),

outlier.shape = 20,

alpha = 0.5

) +

scale_fill_manual(values = colors) +

theme_classic() +

stat_compare_means(

comparisons = list(c("tumor", "normal")),

label = "p.signif",

method = "wilcox.test",

hide.ns = FALSE,

label.x.npc = "center"

) + theme(legend.position = "none")

P4 <- ggplot(data, aes(x = group, y = TUBB1)) +

labs(y = "gene expression level", x = NULL, title = NULL) +

geom_violin(aes(fill = group), position = position_dodge(0.5), alpha = 0.25) +

geom_boxplot(

aes(fill = group),

position = position_dodge(0.5),

outlier.shape = 20,

alpha = 0.5

) +

scale_fill_manual(values = colors) +

theme_classic() +

stat_compare_means(

comparisons = list(c("tumor", "normal")),

label = "p.signif",

method = "wilcox.test",

hide.ns = FALSE,

label.x.npc = "center"

) + theme(legend.position = "none")

P5 <- ggplot(data, aes(x = group, y = VIM)) +

labs(y = "gene expression level", x = NULL, title = NULL) +

geom_violin(aes(fill = group), position = position_dodge(0.5), alpha = 0.25) +

geom_boxplot(

aes(fill = group),

position = position_dodge(0.5),

outlier.shape = 20,

alpha = 0.5

) +

scale_fill_manual(values = colors) +

theme_classic() +

stat_compare_means(

comparisons = list(c("tumor", "normal")),

label = "p.signif",

method = "wilcox.test",

hide.ns = FALSE,

label.x.npc = "center"

) + theme(legend.position = "none")

combined_plot <- plot_grid(P1, P2, P3, P4, P5, nrow = 1, ncol = 5)

# Save the plot as a PDF file

ggsave(

"tcga-brca_expression.pdf",

combined_plot,

width = 20,

height = 6,

units = "cm"

)

#--------------------------Differential Expression and Enrichment Analysis----------------------------------

# Extract the count matrix for tumor samples

tumor_count <- exp_count_gene_name[, substr(colnames(exp_count_gene_name), 14, 14) == "0"]

colnames(tumor_count) <- substr(colnames(tumor_count), 1, 12)

tumor_count <- tumor_count[, !duplicated(colnames(tumor_count))]

# Prepare grouping information

group <- data.frame(Patients = riskout$Patients, group = riskout$riskgroup)

temp <- tumor_count[, colnames(tumor_count) %in% group$Patients]

temp1 <- data.frame(Patients = colnames(temp), No = c(1:ncol(temp)))

temp1 <- merge(temp1, group, by = "Patients", sort = FALSE)

group <- as.factor(temp1$group)

table(group)

design <- model.matrix(~0 + group)

colnames(design) <- levels(group)

row.names(design) <- temp1$Patients

# Differential expression analysis

DGElist <- DGEList(counts = temp, group = group)

# Normalization

DGElist <- calcNormFactors(DGElist)

# Convert data to logCPM and fit a linear model

v <- voom(DGElist, design, plot = TRUE, normalize = "quantile") # logCPM conversion

fit <- lmFit(v, design) # Fit linear model

constrasts <- paste(rev(levels(group)), collapse = "-")

cont.matrix <- makeContrasts(contrasts = constrasts, levels = design)

fit2 <- contrasts.fit(fit, cont.matrix)

fit2 <- eBayes(fit2)

DEG <- topTable(fit2, coef = constrasts, n = Inf) # Extract a table of the top-ranked genes from a linear model fit.

DEG <- na.omit(DEG) # Remove rows with missing values on columns specified

fdr <- 0.05

k1 <- (DEG$adj.P.Val < fdr) & (DEG$logFC < (-1))

k2 <- (DEG$adj.P.Val < fdr) & (DEG$logFC > (1))

change <- ifelse(k1, "down", ifelse(k2, "up", "stable"))

DEG$change <- change

nrow(DEG[DEG$change != "stable", ]) # Number of differentially expressed genes: 818

save(DEG, file = "DEG_risk.rda")

load("DEG_risk.rda")

#---Volcano Plot---

pdf(file = "vol_groups.pdf", height = 5, width = 5)

x_lim <- max(DEG$logFC, -DEG$logFC)

vol_cluster <- ggplot(DEG, aes(logFC, -1 * log10(adj.P.Val), color = change)) +

geom_point(size = 1) +

theme_bw() +

xlim(-x_lim, x_lim) +

labs(x = "log2(FC)", y = "-log10(adjP)") +

scale_color_manual(values = c("#A52A2A", "grey", "#21766d")) +

geom_hline(aes(yintercept = -1 * log10(0.05)), color = "black", linetype = "dashed") +

geom_vline(xintercept = c(-1, 1), color = "black", linetype = "dashed")

print(vol_cluster)

dev.off()

# No significantly differentially expressed genes, so perform GSEA analysis

library(clusterProfiler)

library(org.Hs.eg.db)

library(stringr)

library(enrichplot)

DEG$SYMBOL <- rownames(DEG)

gseaDATA <- DEG[, c("logFC", "SYMBOL")]

# ID conversion

gene <- bitr(gseaDATA[, 2],

fromType = "SYMBOL",

toType = "ENTREZID",

OrgDb = "org.Hs.eg.db")

# Merge data

gseaDATA <- as.data.frame(gseaDATA)

gseaDATA <- gseaDATA[(gseaDATA$SYMBOL) %in% (gene$SYMBOL), ]

gseaDATA <- merge(gseaDATA, gene, by = "SYMBOL")

gseaDATA <- gseaDATA[order(gseaDATA$logFC, decreasing = TRUE), ]

gsea <- gseaDATA$logFC

names(gsea) <- gseaDATA$ENTREZID

head(gsea)

GO <- gseGO(

gsea, # gsea

ont = "ALL", # "BP", "MF", "CC", or "ALL"

OrgDb = org.Hs.eg.db, # Human annotation genes

keyType = "ENTREZID",

pvalueCutoff = 0.05,

pAdjustMethod = "BH" # p-value adjustment method

)

kegg <- gseKEGG(

gsea,

organism = "hsa",

keyType = "kegg",

exponent = 1,

minGSSize = 10,

maxGSSize = 500,

eps = 1e-10,

pvalueCutoff = 0.05,

pAdjustMethod = "BH",

verbose = TRUE,

use_internal_data = FALSE,

seed = FALSE,

by = "fgsea"

)

library(ggplot2)

dotplot(GO, split = ".sign", color = "pvalue", showCategory = 8) + facet_grid(~.sign) # Plot points by p-value

dotplot(kegg, split = ".sign", color = "pvalue", showCategory = 8) + facet_grid(~.sign) # Plot points by p-value

gseaplot2(kegg, 1:5, pvalue_table = TRUE)

gseaplot2(GO, 1:5, pvalue_table = TRUE)

ridgeplot(GO)

save(GO, kegg, file = "gsea.rda")
